# Supplementary material for: Machine learning meets complex networks via coalescent embedding in the hyperbolic space
Source: Nat Commun. 2017 Nov 20;8:1615. doi: 10.1038/s41467-017-01825-5 (PMC5694768; doi:10.1038/s41467-017-01825-5)
Supplement: Supplementary file 1 — Supplementary Information [file 41467_2017_1825_MOESM1_ESM.pdf]

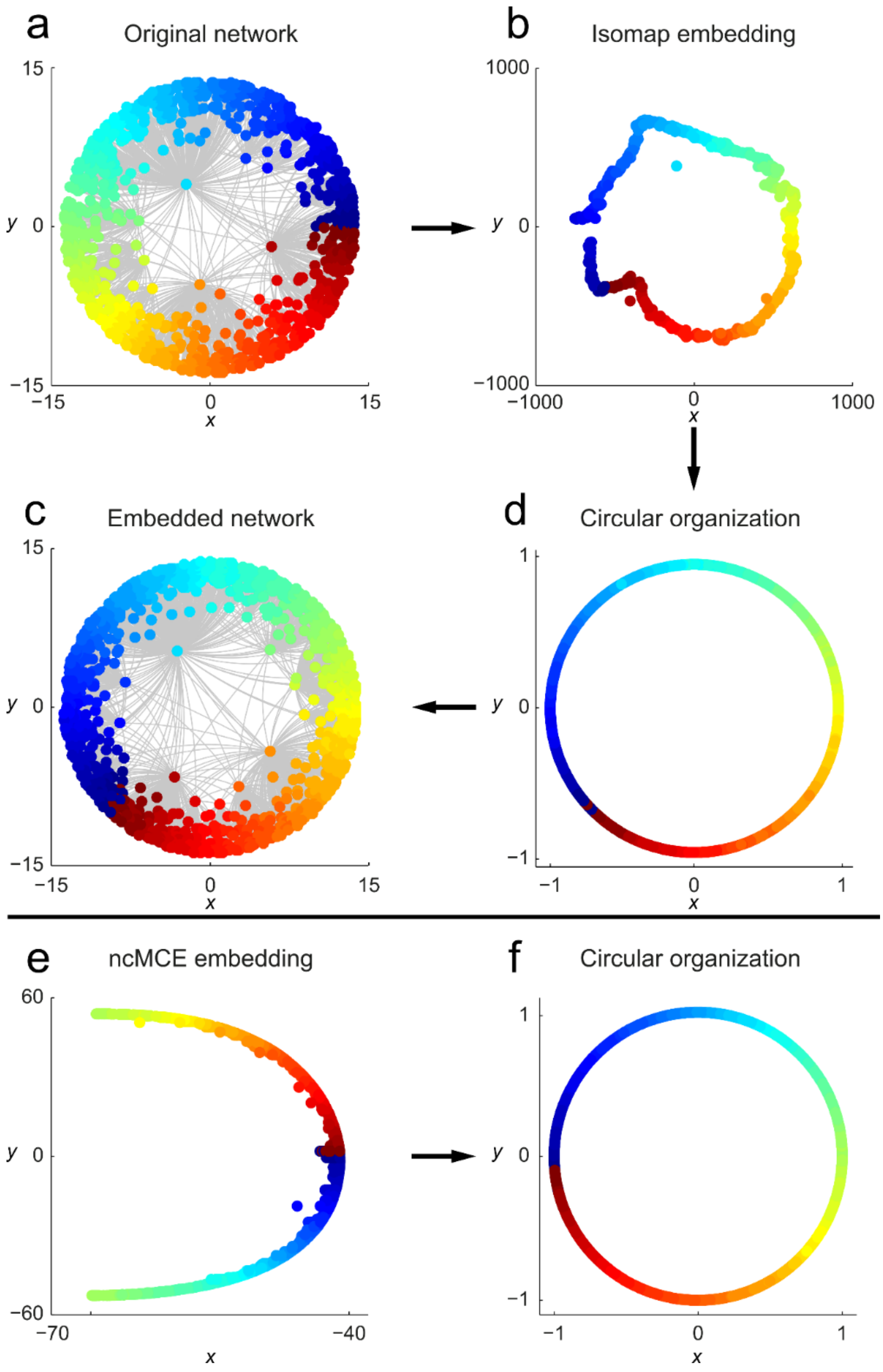

Supplementary Figure 1. Coalescent embedding with RA pre-weighting

(a) We show the original synthetic network generated by the PSO model in the hyperbolic space. (b) The Isomap algorithm (ISO) starting from the adjacency matrix pre-weighted with the repulsion-attraction (RA) rule offers an embedding of the network nodes that is organized according to a circular pattern that follows the angular coordinates of the original PSO model. The circular pattern is visible more clearly compared to the embedding without the pre-weighting (Fig. 1). (d) The nodes are projected over a circumference and adjusted equidistantly according to the step 3.2 of the algorithm described in Methods. (c) The radial coordinates are given according to Equation 4. (e) A different pattern is obtained for an algorithm named ncMCE. The circular pattern is linearized and the nodes are ordered along the second dimension of embedding according to their similarities. (f) If we accommodate the node points on the circumference following the same ordering as the second dimension of embedding, we can again recover an unbroken circular pattern that resembles the angular coordinates of the original PSO model.

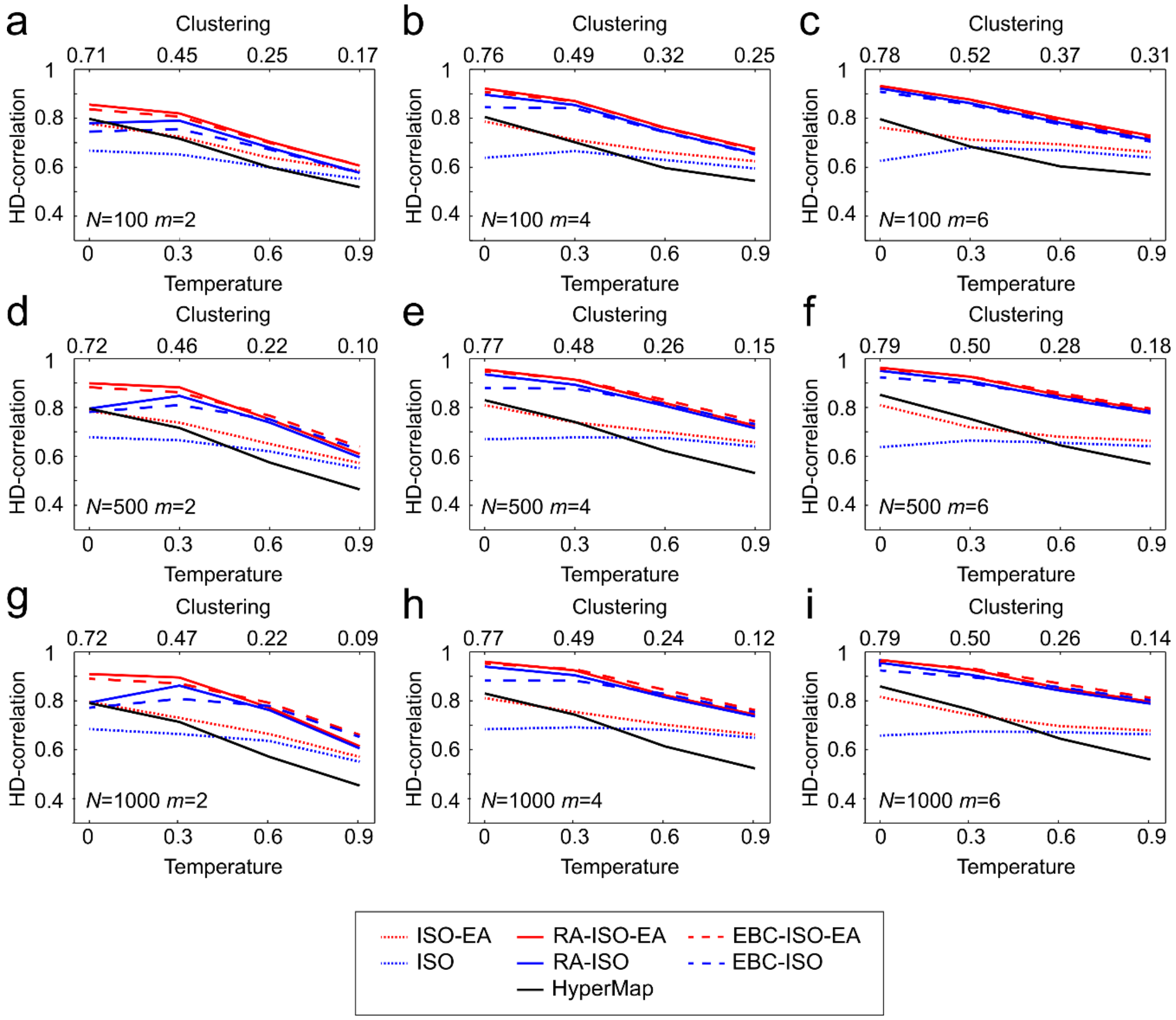

Supplementary Figure 2. HD-correlation on PSO synthetic networks for ISO
(a-i) The figure is equivalent to Fig. 3, but all the variants of ISO are compared.

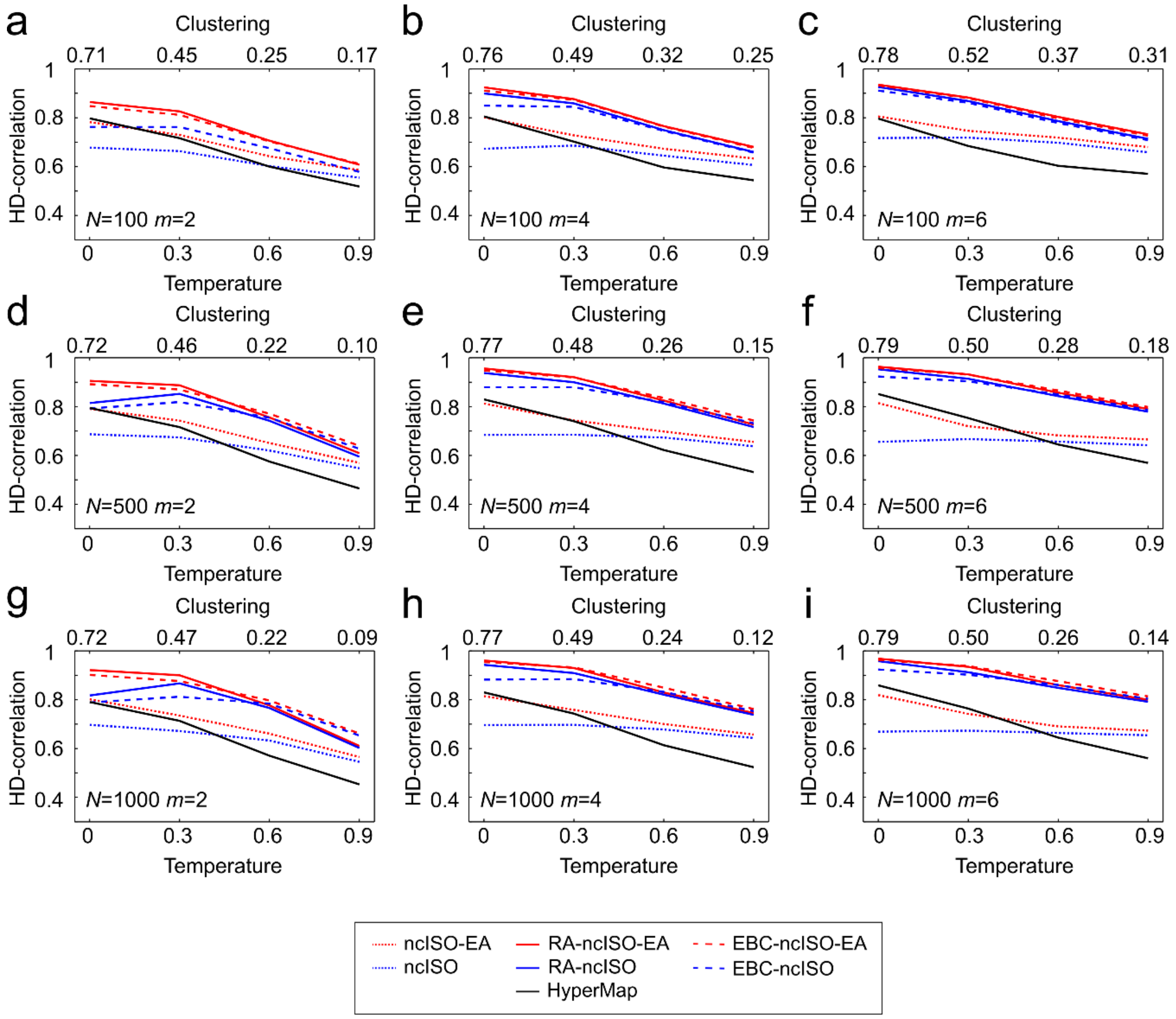

Supplementary Figure 3. HD-correlation on PSO synthetic networks for ncISO

(a-i) The figure is equivalent to Fig. 3, but all the variants of ncISO are compared.

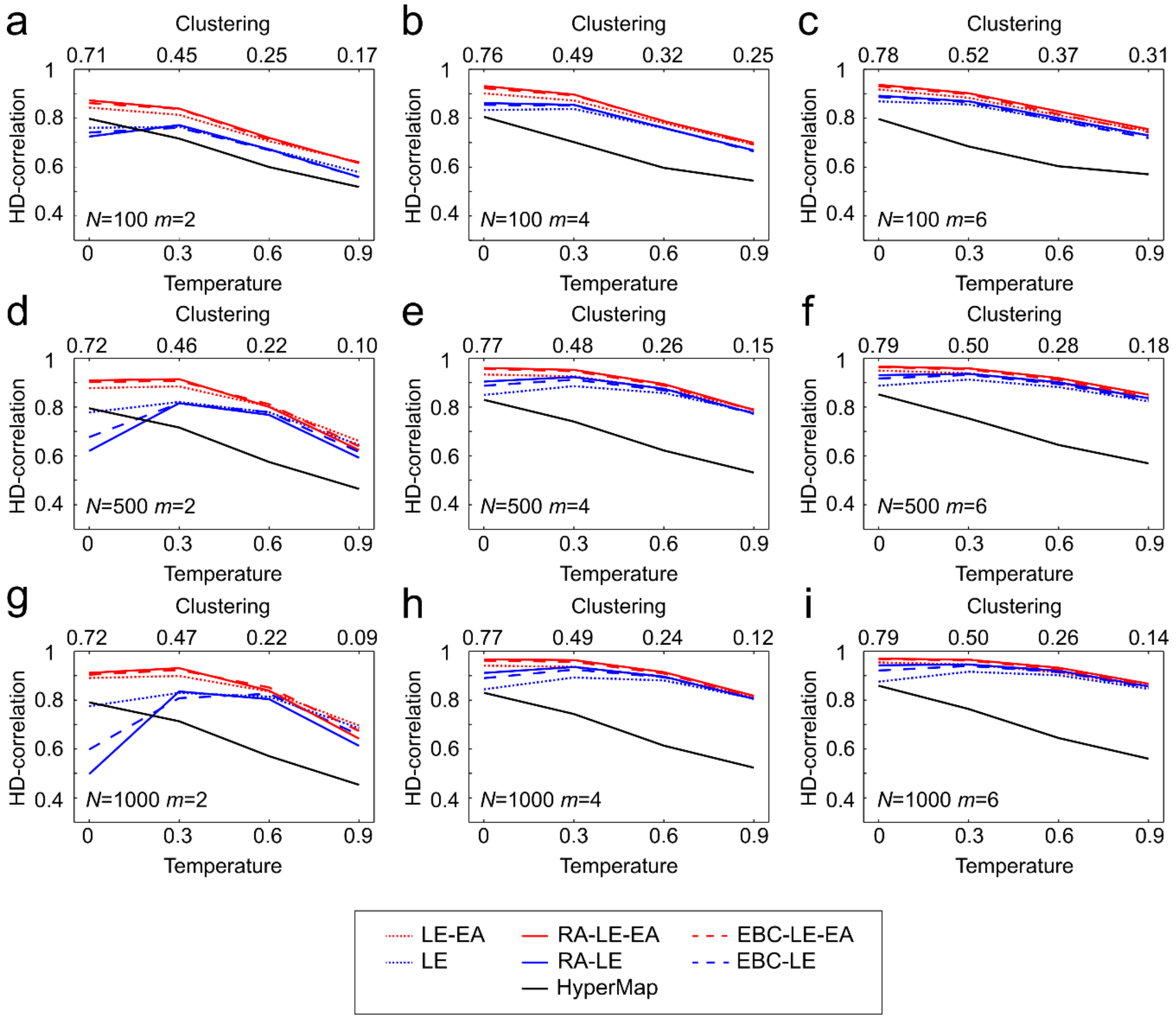

Supplementary Figure 4. HD-correlation on PSO synthetic networks for LE

(a-i) The figure is equivalent to Fig. 3, but all the variants of LE are compared.

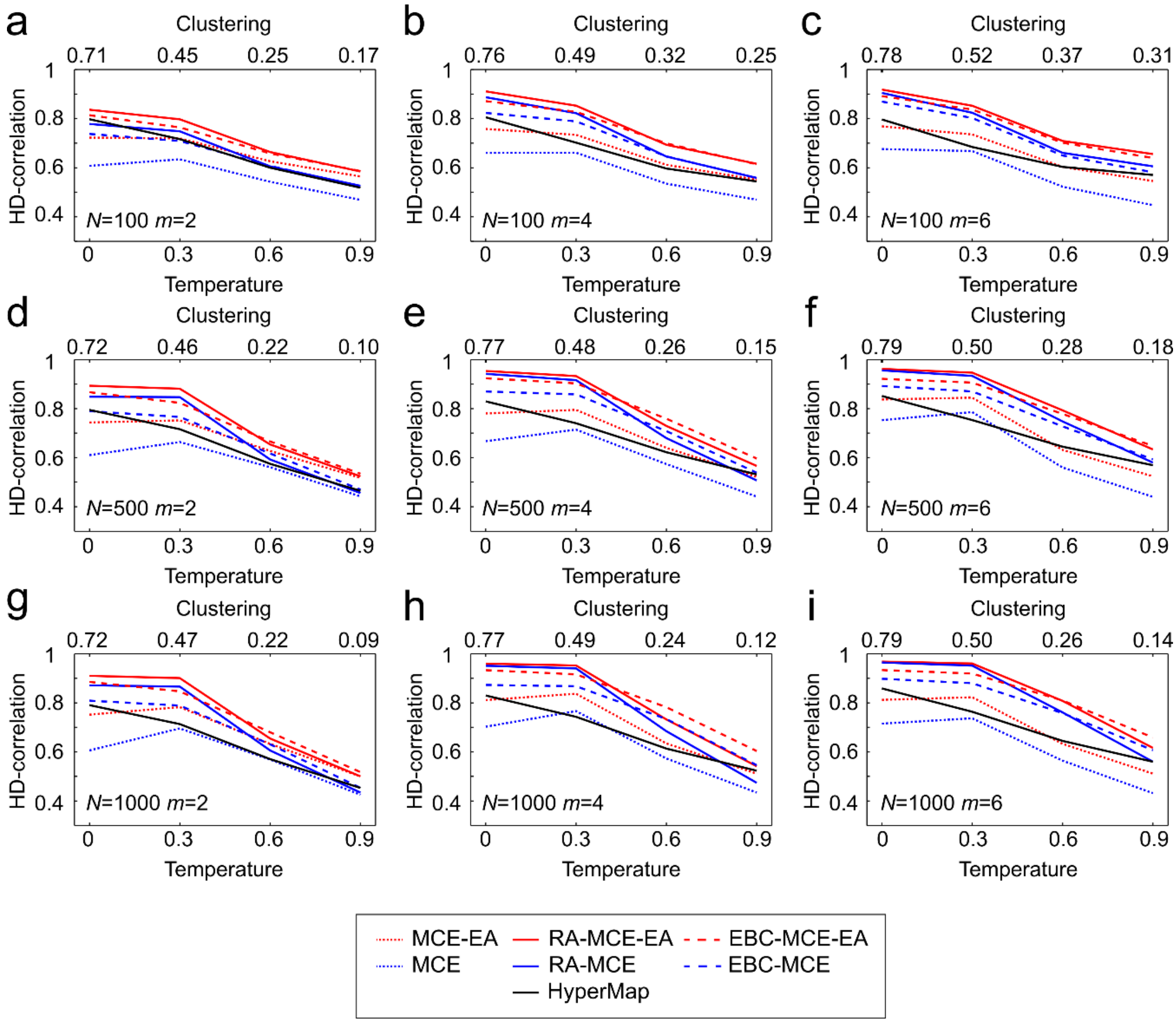

Supplementary Figure 5. HD-correlation on PSO synthetic networks for MCE

(a-i) The figure is equivalent to Fig. 3, but all the variants of MCE are compared.

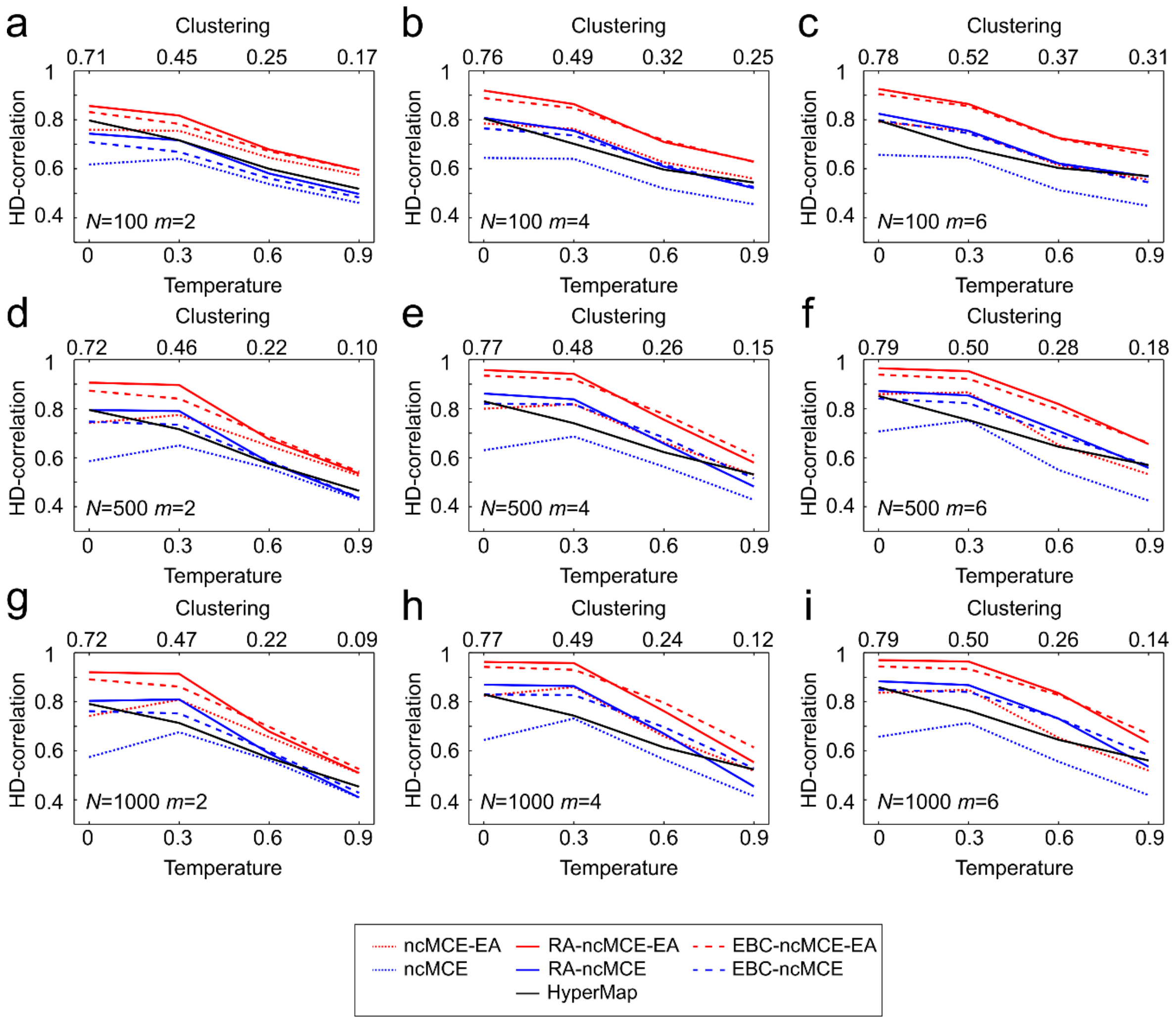

Supplementary Figure 6. HD-correlation on PSO synthetic networks for ncMCE

(a-i) The figure is equivalent to Fig. 3, but all the variants of ncMCE are compared.

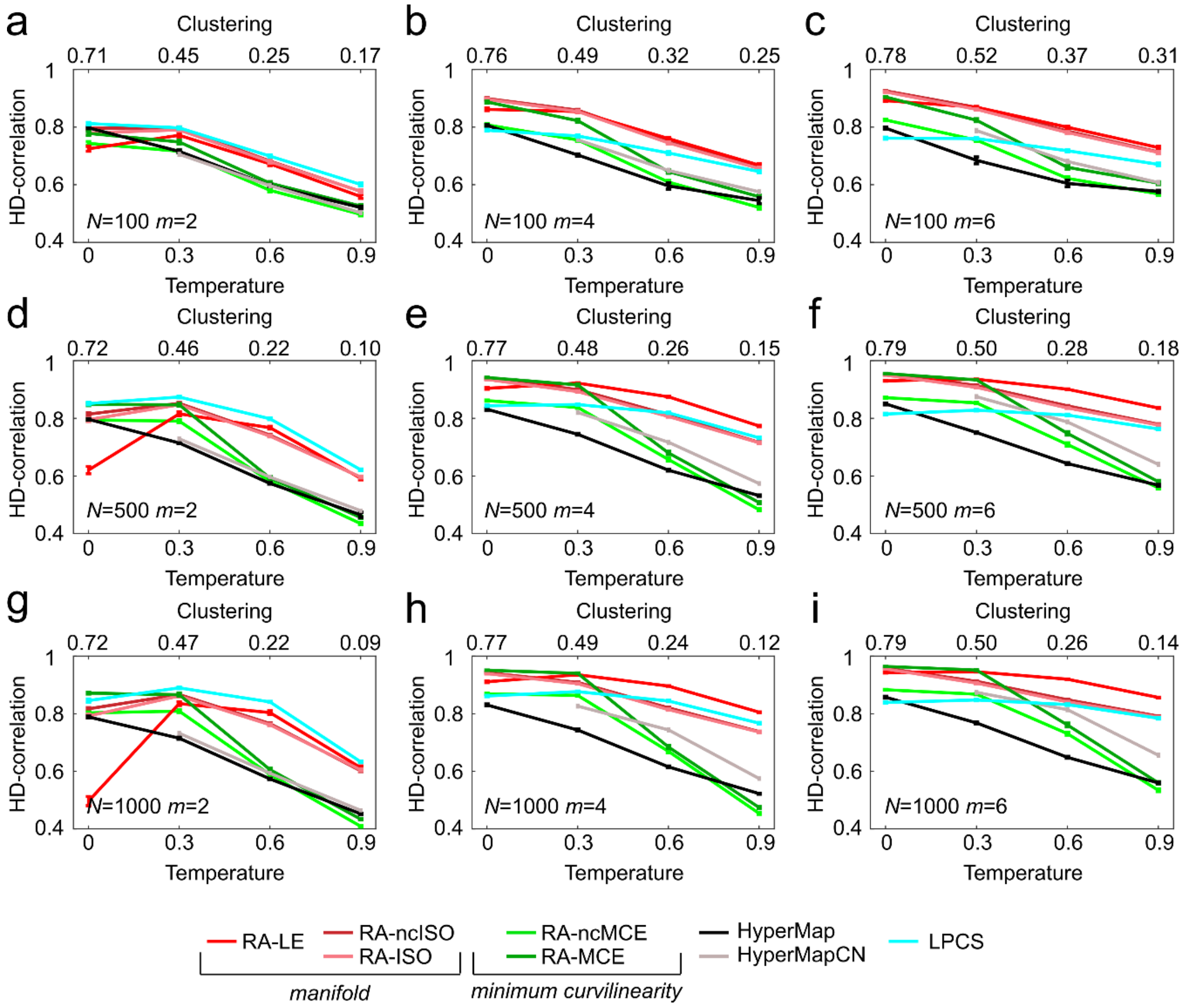

Supplementary Figure 7. HD-correlation on PSO synthetic networks

(a-i) The figure is equivalent to Fig. 3, but the methods without EA are shown. Comparing the two figures, it is evident that the ability of EA to adjust for the local positional noise makes a difference. In fact, when $m = 2$ and temperatures are low, RA-LE without equidistant adjustment suffers a strong performance reduction in comparison to the case in which EA is applied.

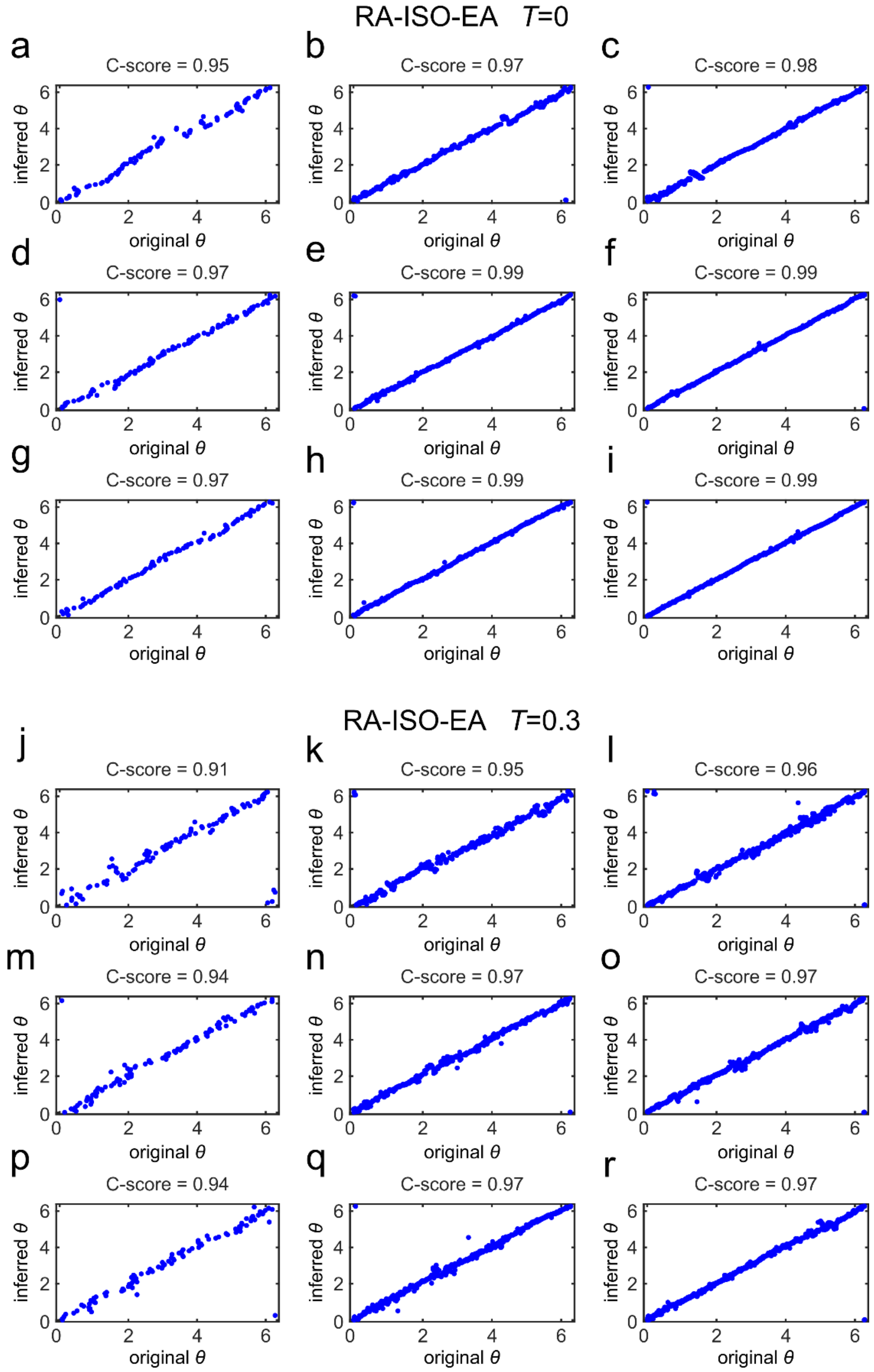

Supplementary Figure 8. Angular coordinates comparison for RA-ISO-EA (T = 0, T = 0.3)

For all the combinations of the PSO parameters N (size) and m (half of average degree), fixing T = 0 (a-i) and T = 0.3 (j-r), we chose among the synthetic networks embedded with RA-ISO-EA the ones with the best C-score. For these networks we plotted the aligned inferred angular coordinates against the original angular coordinates as described in Fig. 4.

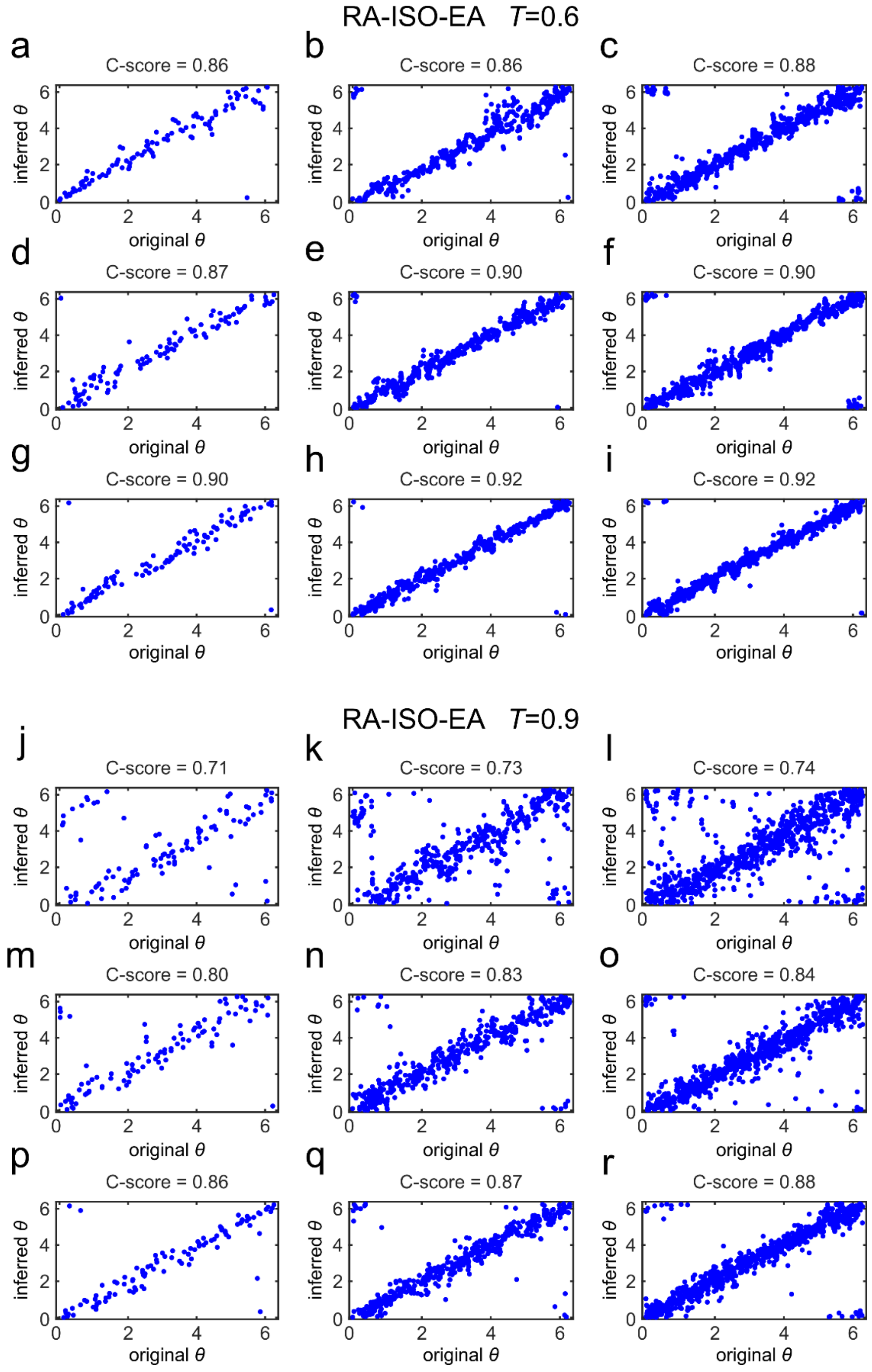

Supplementary Figure 9. Angular coordinates comparison for RA-ISO-EA (*T* = 0.6, *T* = 0.9)

For all the combinations of the PSO parameters *N* (size) and *m* (half of average degree), fixing *T* = 0.6 (a-i) and *T* = 0.9 (j-r), we chose among the synthetic networks embedded with RA-ISO-EA the ones with the best C-score. For these networks we plotted the aligned inferred angular coordinates against the original angular coordinates as described in Fig. 4.

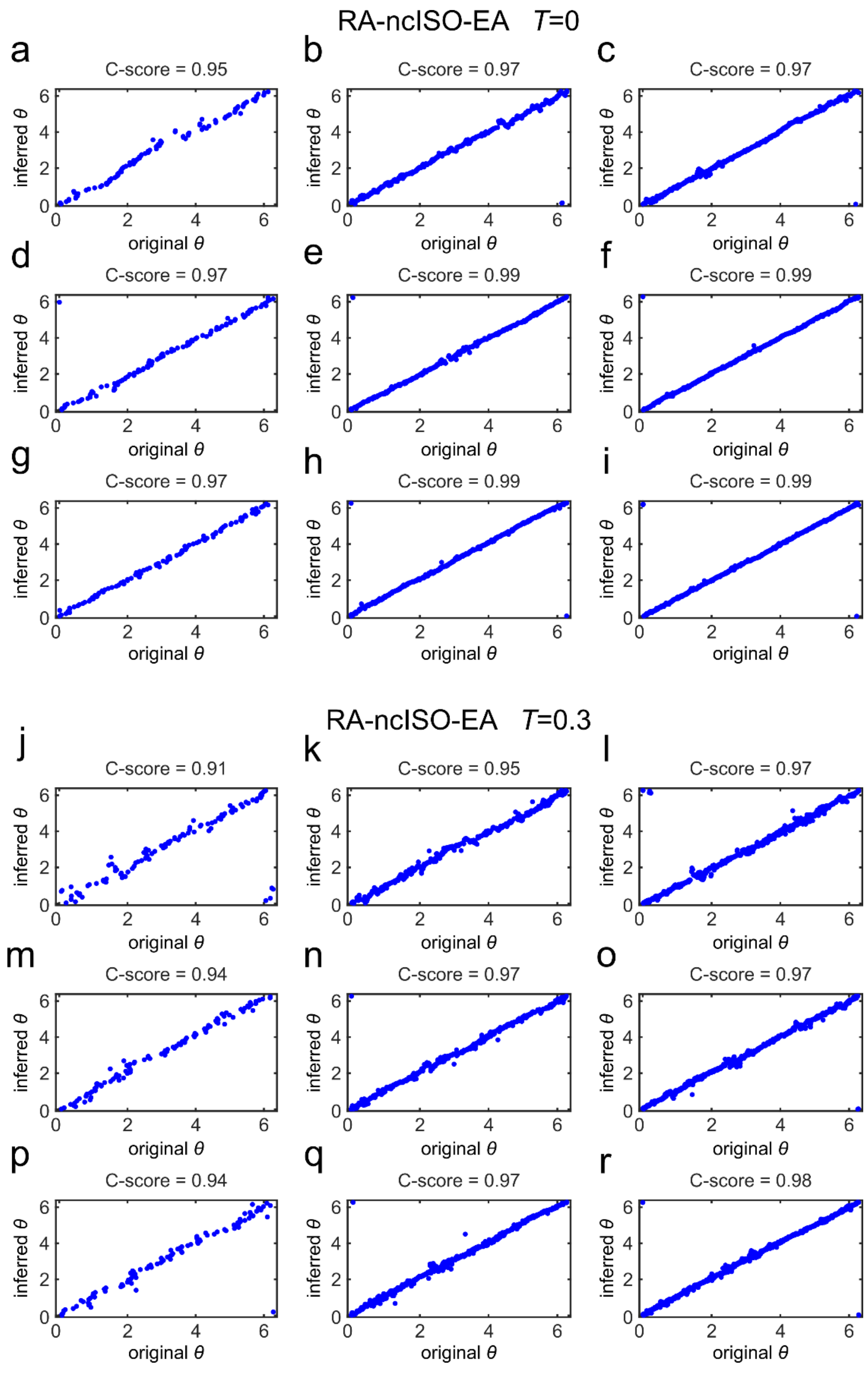

Supplementary Figure 10. Angular coordinates comparison for RA-ncISO-EA (T = 0, T = 0.3)
For all the combinations of the PSO parameters N (size) and m (half of average degree), fixing T = 0 (a-i) and T = 0.3 (j-r), we chose among the synthetic networks embedded with RA-ncISO-EA the ones with the best C-score. For these networks we plotted the aligned inferred angular coordinates against the original angular coordinates as described in Fig. 4.

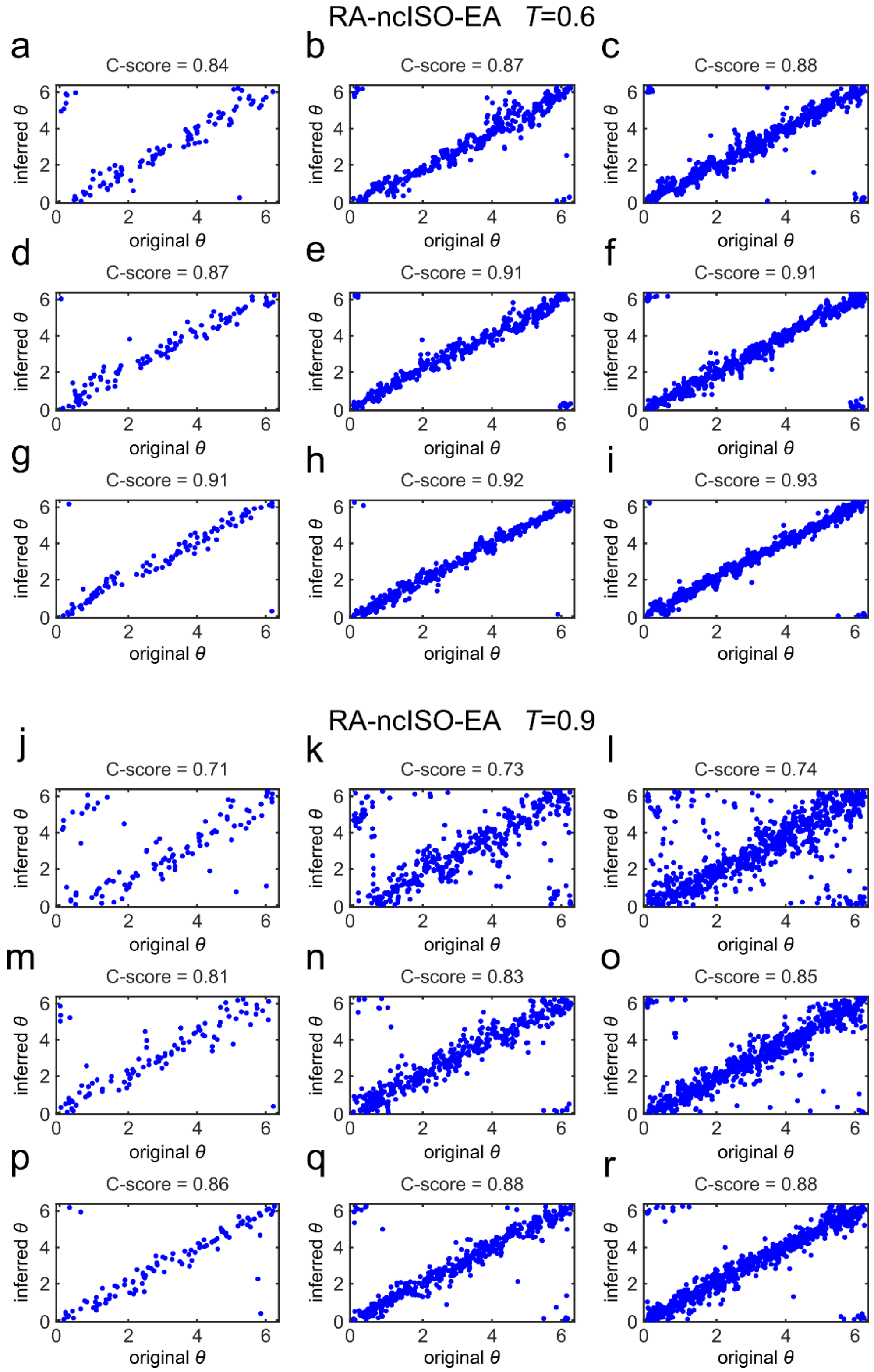

Supplementary Figure 11. Angular coordinates comparison for RA-ncISO-EA (*T* = 0.6, *T* = 0.9)
For all the combinations of the PSO parameters *N* (size) and *m* (half of average degree), fixing *T* = 0.6 (a-i) and *T* = 0.9 (j-r), we chose among the synthetic networks embedded with RA-ncISO-EA the ones with the best C-score. For these networks we plotted the aligned inferred angular coordinates against the original angular coordinates as described in Fig. 4.

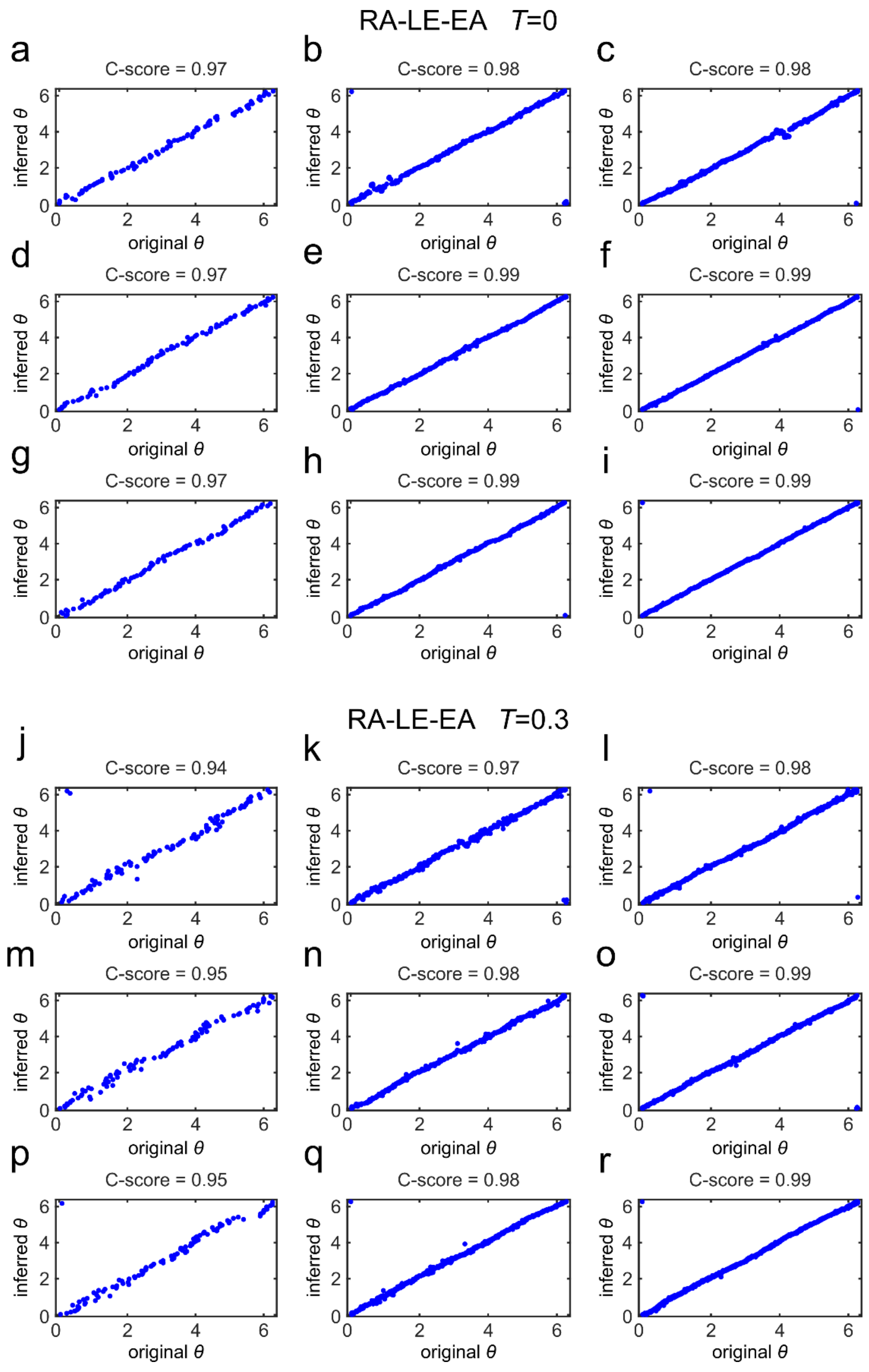

Supplementary Figure 12. Angular coordinates comparison for RA-LE-EA (T = 0, T = 0.3)

For all the combinations of the PSO parameters N (size) and m (half of average degree), fixing T = 0 (a-i) and T = 0.3 (j-r), we chose among the synthetic networks embedded with RA-LE-EA the ones with the best C-score. For these networks we plotted the aligned inferred angular coordinates against the original angular coordinates as described in Fig. 4.

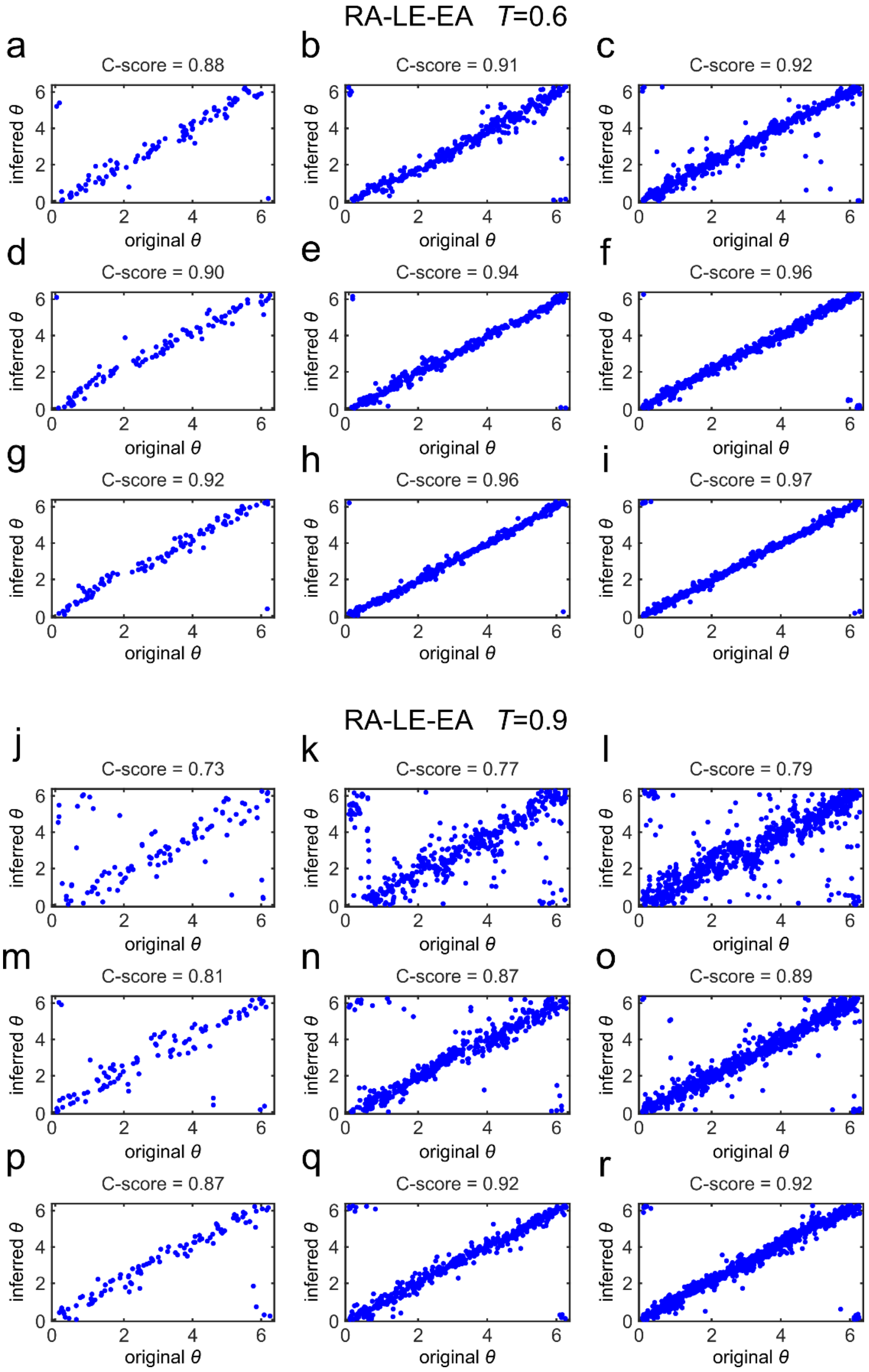

Supplementary Figure 13. Angular coordinates comparison for RA-LE-EA (T = 0.6, T = 0.9)

For all the combinations of the PSO parameters N (size) and m (half of average degree), fixing T = 0.6 (a-i) and T = 0.9 (j-r), we chose among the synthetic networks embedded with RA-LE-EA the ones with the best C-score. For these networks we plotted the aligned inferred angular coordinates against the original angular coordinates as described in Fig. 4.

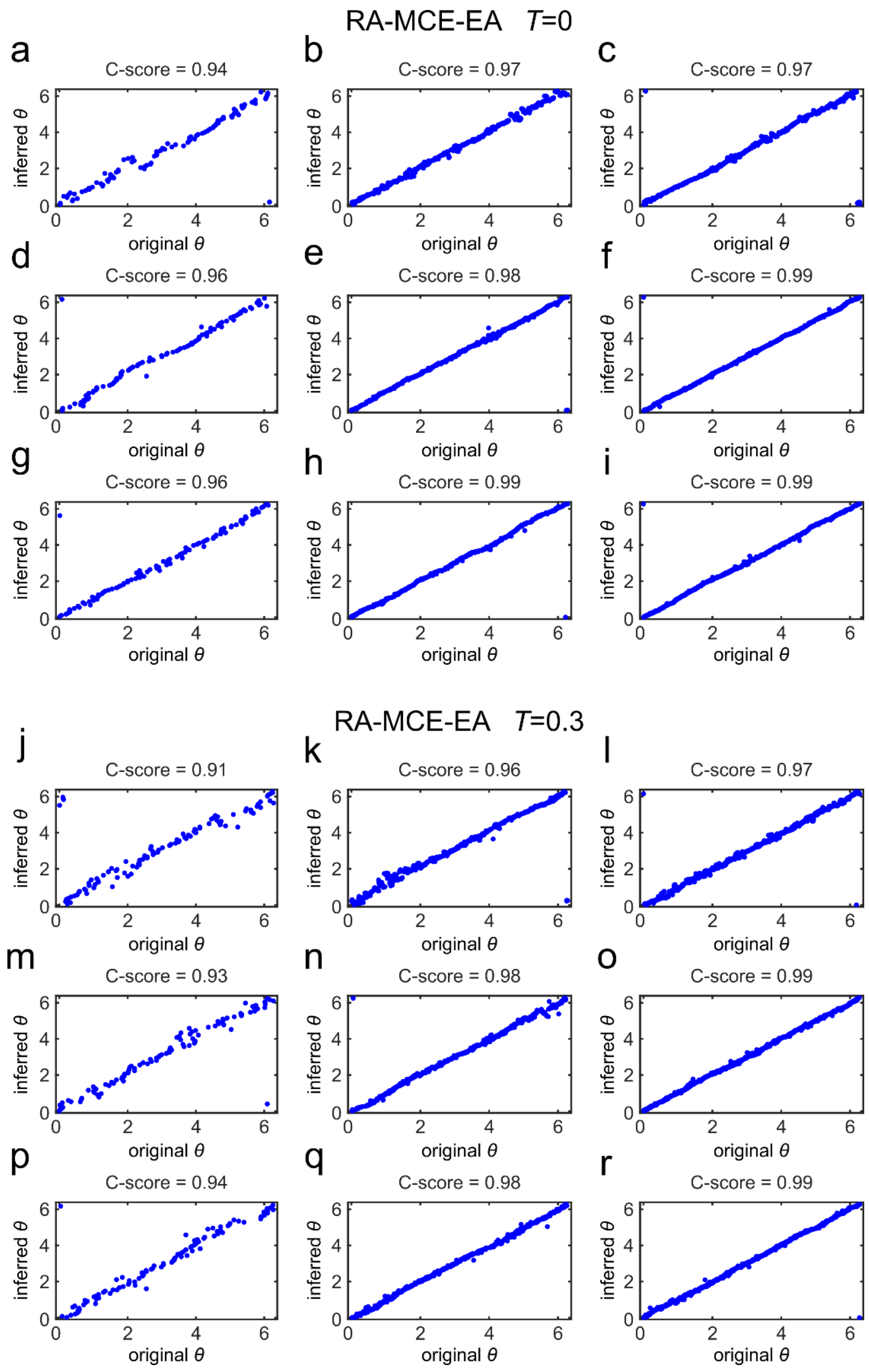

Supplementary Figure 14. Angular coordinates comparison for RA-MCE-EA (T = 0, T = 0.3)

For all the combinations of the PSO parameters N (size) and m (half of average degree), fixing T = 0 (a-i) and T = 0.3 (j-r), we chose among the synthetic networks embedded with RA-MCE-EA the ones with the best C-score. For these networks we plotted the aligned inferred angular coordinates against the original angular coordinates as described in Fig. 4.

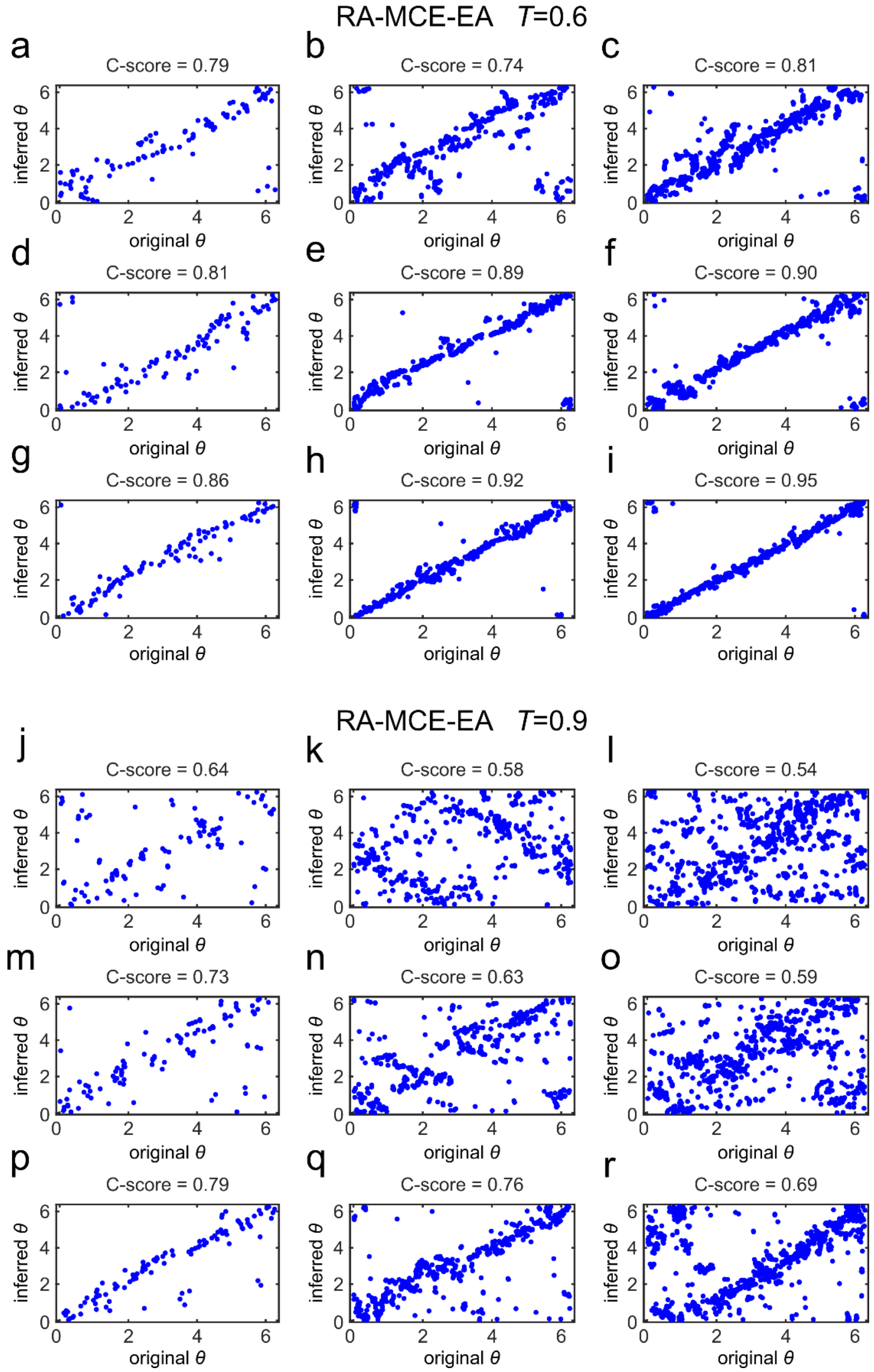

Supplementary Figure 15. Angular coordinates comparison for RA-MCE-EA (*T* = 0.6, *T* = 0.9)

For all the combinations of the PSO parameters *N* (size) and *m* (half of average degree), fixing *T* = 0.6 (a-i) and *T* = 0.9 (j-r), we chose among the synthetic networks embedded with RA-MCE-EA the ones with the best C-score. For these networks we plotted the aligned inferred angular coordinates against the original angular coordinates as described in Fig. 4.

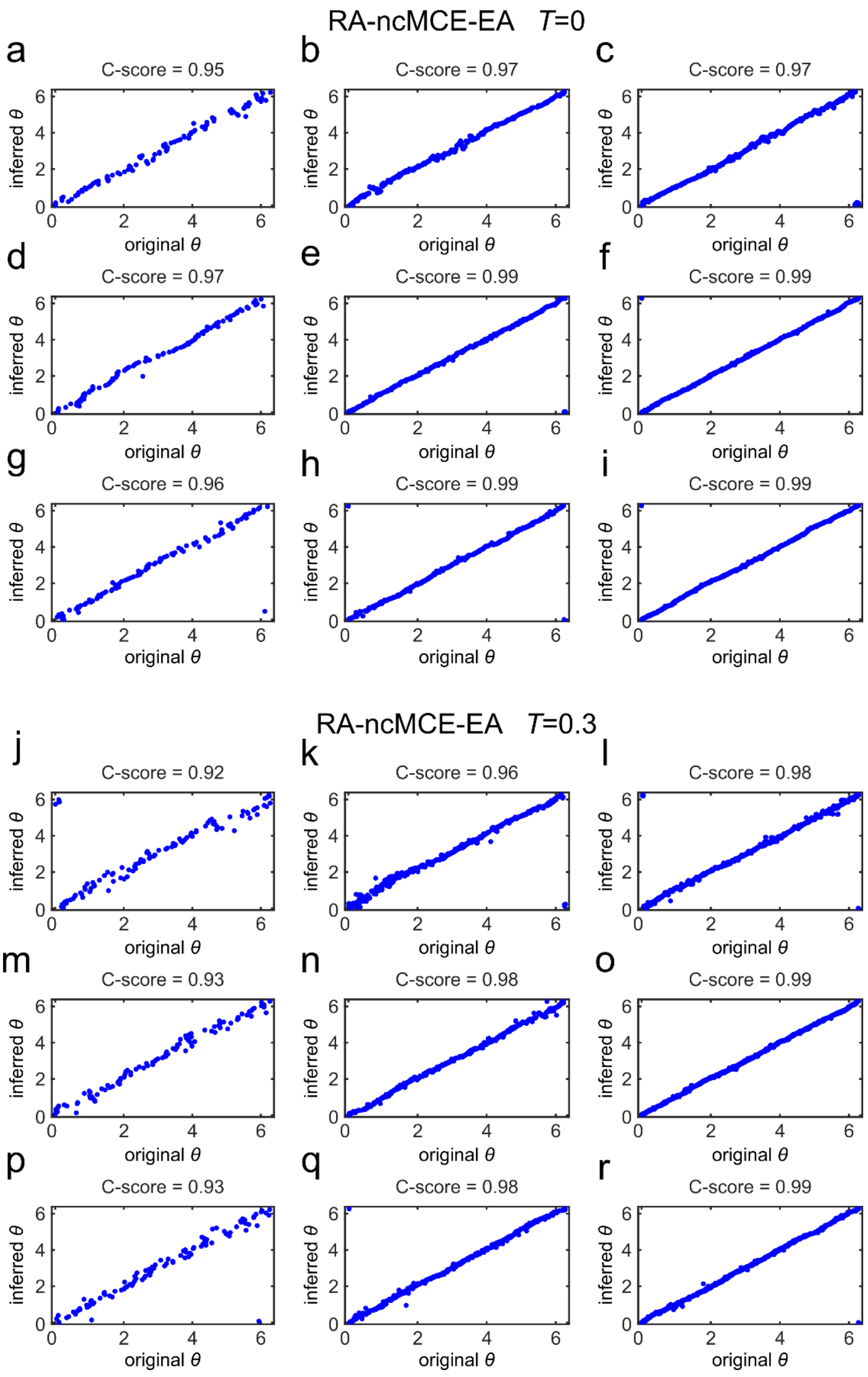

Supplementary Figure 16. Angular coordinates comparison for RA-ncMCE-EA (T = 0, T = 0.3)

For all the combinations of the PSO parameters N (size) and m (half of average degree), fixing T = 0 (a-i) and T = 0.3 (j-r), we chose among the synthetic networks embedded with RA-ncMCE-EA the ones with the best C-score. For these networks we plotted the aligned inferred angular coordinates against the original angular coordinates as described in Fig. 4.

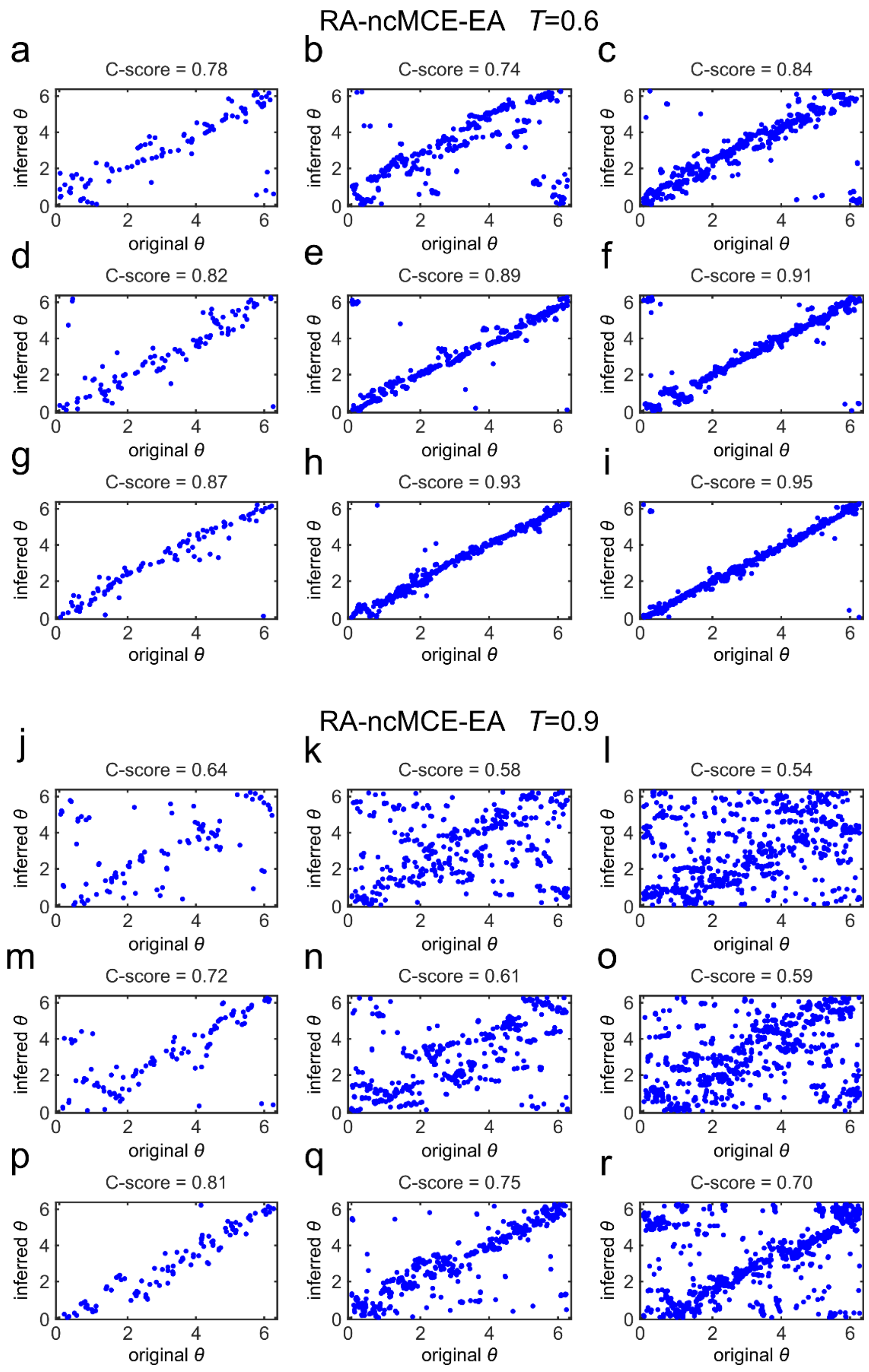

Supplementary Figure 17. Angular coordinates comparison for RA-ncMCE-EA (*T* = 0.6, *T* = 0.9)
For all the combinations of the PSO parameters *N* (size) and *m* (half of average degree), fixing *T* = 0.6 (a-i) and *T* = 0.9 (j-r), we chose among the synthetic networks embedded with RA-ncMCE-EA the ones with the best C-score. For these networks we plotted the aligned inferred angular coordinates against the original angular coordinates as described in Fig. 4.

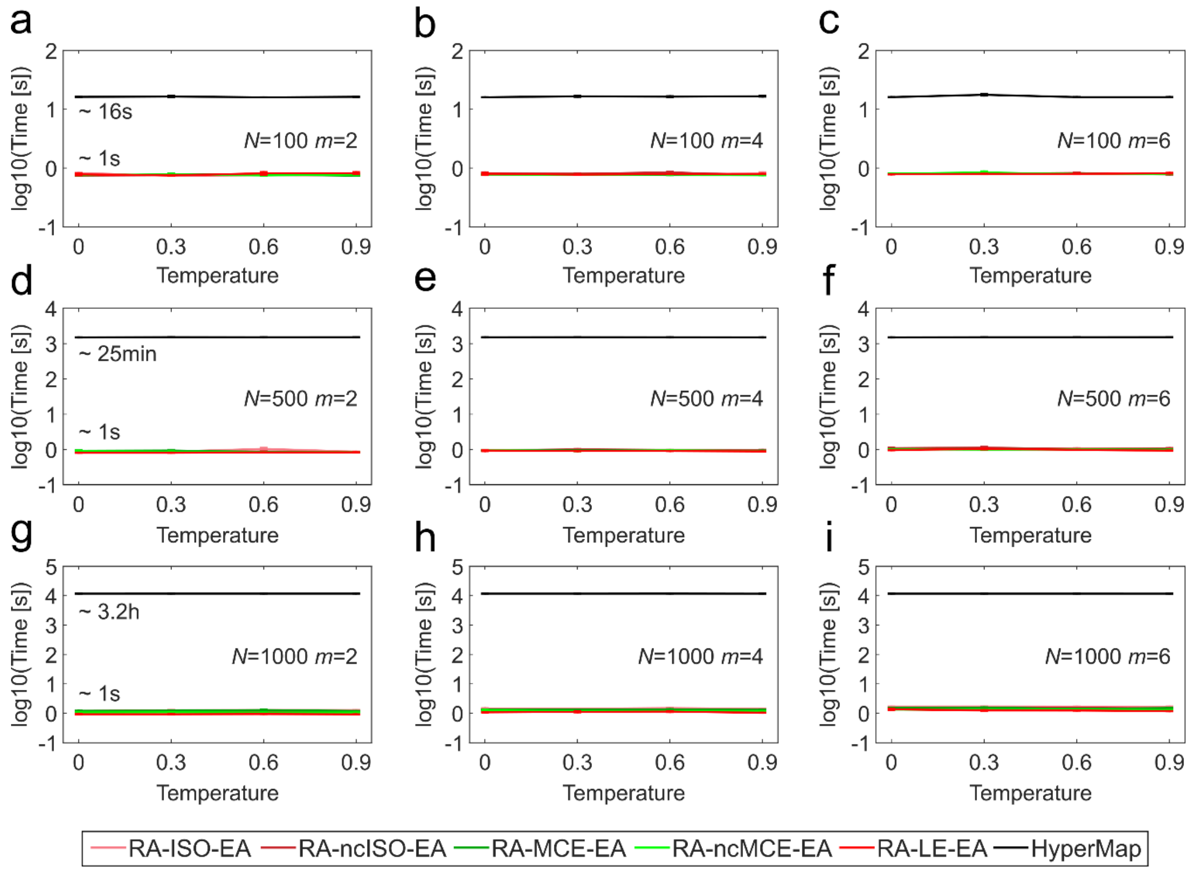

Supplementary Figure 18. Time on PSO synthetic networks

(a-i) For the same PSO networks in Fig. 3, the computational time shows the large efficiency of the coalescent embedding based approaches that generally required around one second to embed networks with 1000 nodes, while HyperMap spent approximately 3 hours for the same task (software and hardware details in the Methods).

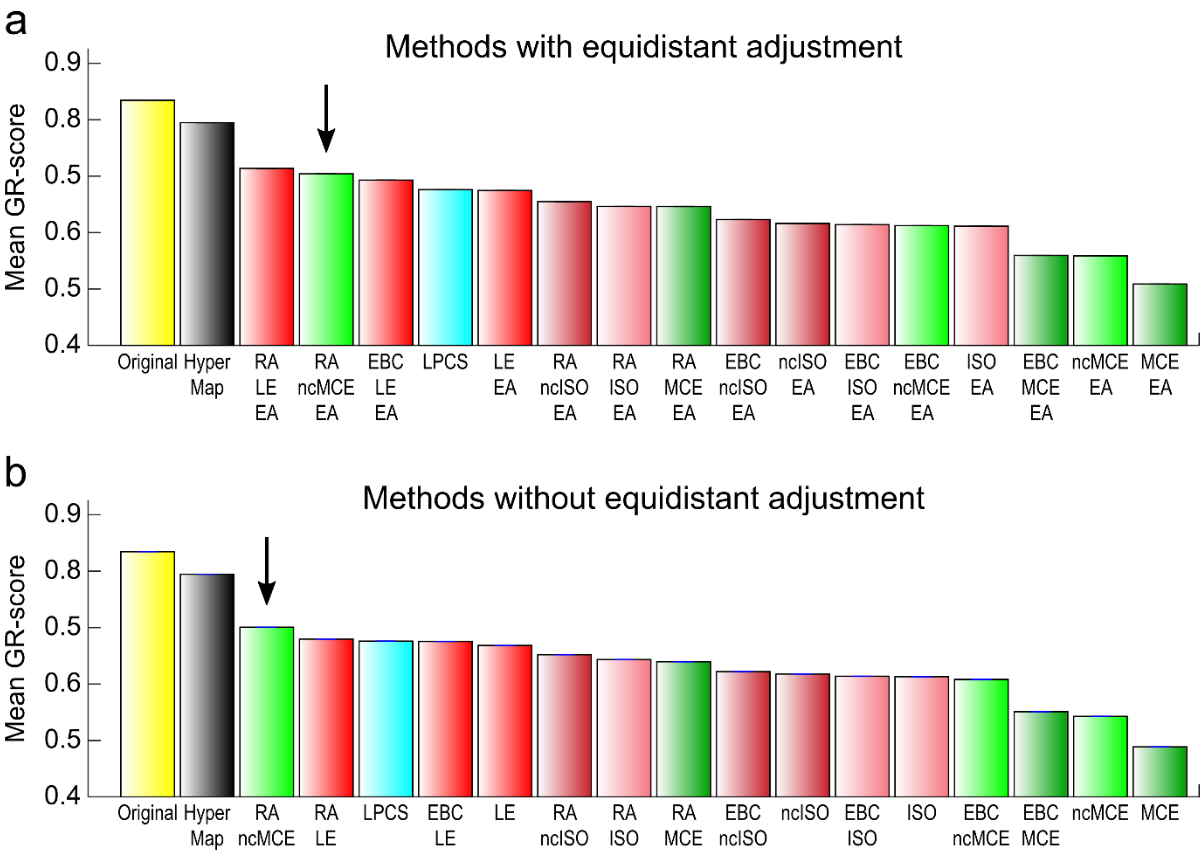

Supplementary Figure 19. Greedy routing on PSO synthetic networks

The same PSO networks considered in Fig. 3 have been mapped using the hyperbolic embedding techniques and the greedy routing in the geometrical space has been evaluated. The barplot reports for each method the mean GR-score over all the PSO parameter combinations. The GR-score is a metric to evaluate the efficiency of the greedy routing, which assumes values between 0, when all the routings are unsuccessful, and 1, when all the packets reach the destination through the shortest path (see Methods for details). Both the EA (a) and non-EA (b) variants are reported, in order to check whether the equidistant adjustment might affect the navigability. The score for HyperMap-CN is not reported since the value for $T = 0$ is missing, because the original code assumes $T > 0$. The GR-score evaluated over the original coordinates of the PSO model is also shown. A black arrow points the coalescent embedding algorithm RA-ncMCE, as in Fig. 6.

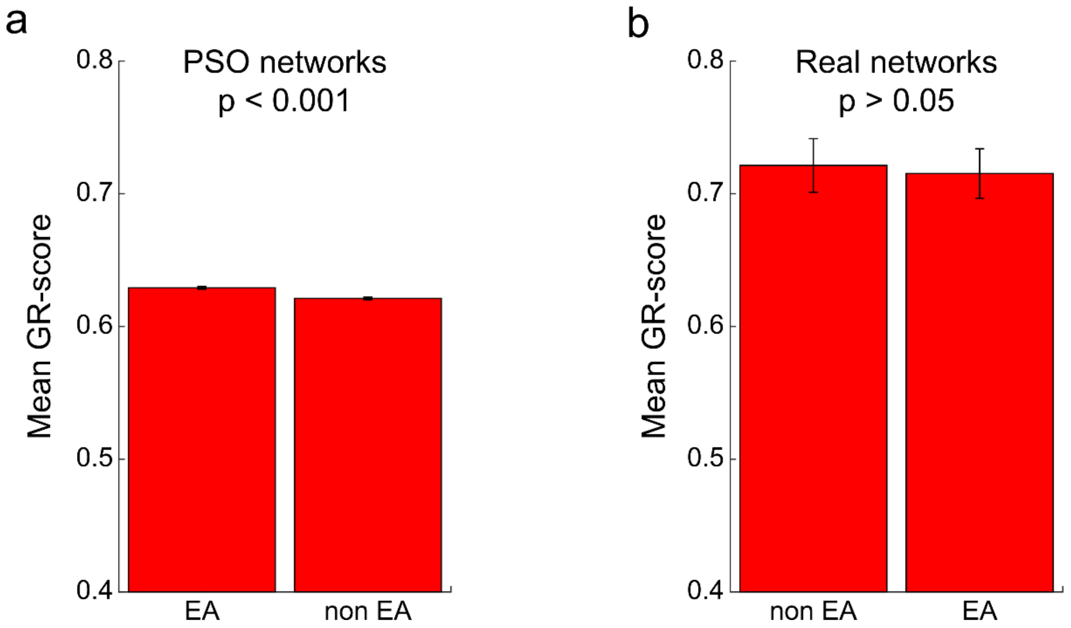

Supplementary Figure 20. Comparison of EA and non-EA for greedy routing

(a) The same PSO networks considered in Fig. 3 have been mapped using the coalescent embedding techniques and the greedy routing in the geometrical space has been evaluated. The barplot reports the GR-score averaged not only over all the PSO parameter combinations but also respectively over all the EA and non-EA coalescent embedding techniques. The corresponding standard error are also shown. The p-value of the permutation test for the mean (10000 iterations) is reported, performed considering the two vectors of GR-scores for the EA and non-EA methods. (b) The 8 real networks whose statistics are reported in Table 1 have been mapped using the coalescent embedding techniques and the greedy routing in the geometrical space has been evaluated. The barplot reports the GR-score averaged not only over the networks but also respectively over all the EA and non-EA coalescent embedding techniques. The corresponding standard error are also shown. The p-value of the permutation test for the mean (10000 iterations) is reported, performed considering the two vectors of GR-scores for the EA and non-EA methods.

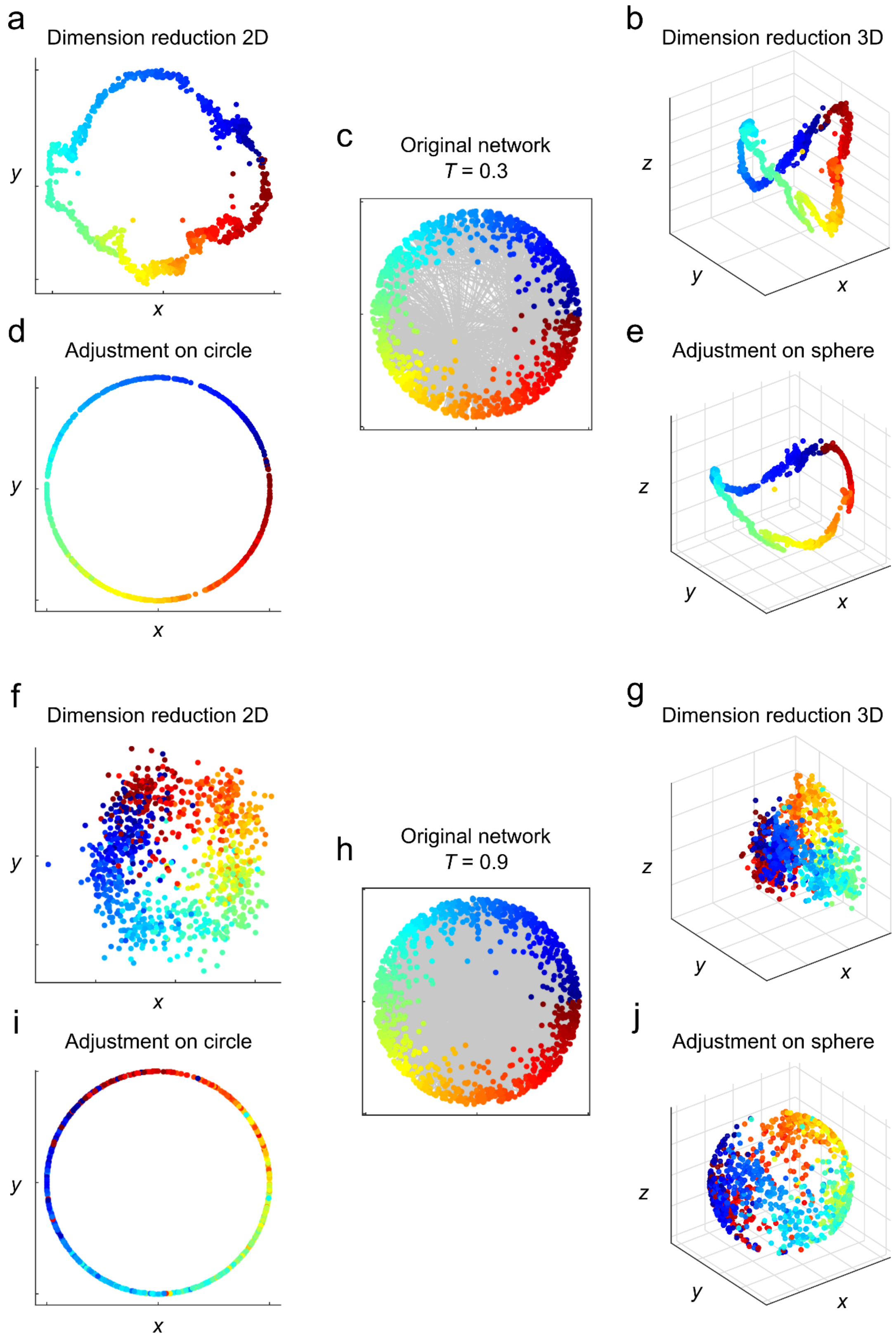

Supplementary Figure 21. Comparison of 2D and 3D embedding for increasing temperature

The figure shows how the similarities of the original PSO network (N = 1000, m = 6, γ = 2.5) are recovered either embedding in 2D (a, f) and arranging the angular coordinates over the circumference of a circle (d, i) or embedding in 3D (b, g) and adjusting the angular coordinates over a sphere (e, j). The figure reports the plots for the temperatures T = 0.3 (a-e) and T = 0.9 (f-j) in order to integrate the Fig. 8, where T = 0 and T = 0.6 are shown.

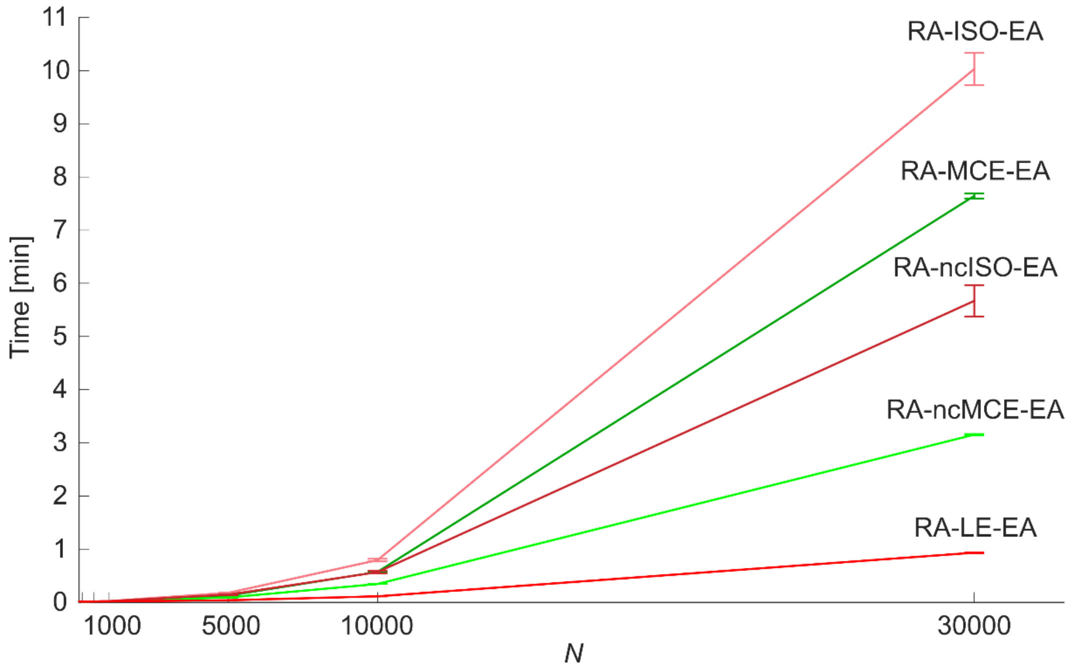

Supplementary Figure 22. Time estimation for coalescent embedding methods on PSO networks

For the PSO parameters $\gamma = 2.5$, $T = 0.3$ (since Supplementary Fig. 4 showed that the time is T invariant) and m = [2, 4, 6], we generated 10 networks of size N = [100, 500, 1000, 5000, 10000] and 5 networks of size N = 30000. The plot reports the average embedding time for increasing network size and for each of the coalescent embedding techniques with RA pre-weighting and equidistant adjustment. The corresponding standard error are also shown.

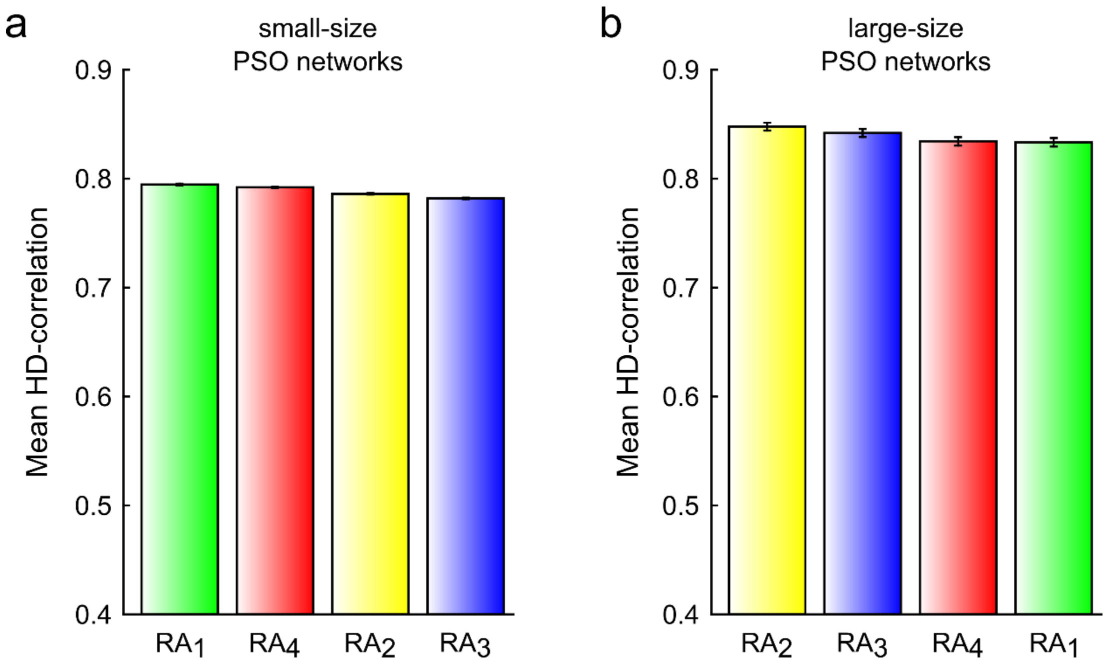

Supplementary Figure 23. Comparison of Repulsion-Attraction (RA) pre-weighting rules

Since there was not a unique possible mathematical formulation of the RA formula, we tested four variants differing in the way in which the degrees of the connected nodes are combined. For each combination of the PSO parameters m = [2, 4, 6] and T = [0, 0.3, 0.6, 0.9], fixing γ = 2.5, we generated 100 networks of size N = [100, 500, 1000], 10 networks of size N = 10000 and 5 networks of size N = 30000. The networks have been embedded using the coalescent embedding techniques applying the different RA pre-weighting variants and the HD-correlation has been evaluated. The figure reports for each RA variant the HD-correlation averaged over the PSO parameter combinations and over the different coalescent embedding techniques, for networks of size N = [100, 500, 1000] (a) and N = [10000, 30000] (b). The corresponding standard error are also shown.

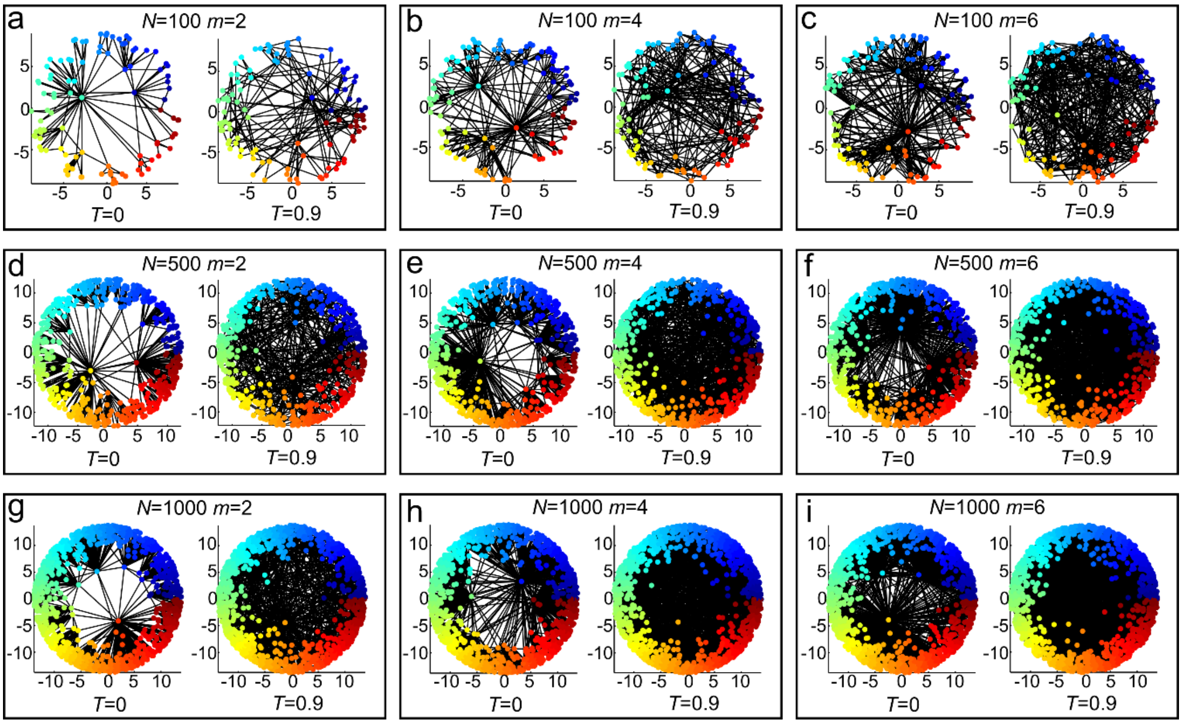

Supplementary Figure 24. Examples of synthetic networks generated by the PSO model

(a-i) For each combination of the parameters N (size) and m (half of average degree), examples of synthetic networks with temperature T = 0 and T = 0.9 are shown, in order to illustrate the increase of randomness and the loss of the tree like structure for high temperatures.

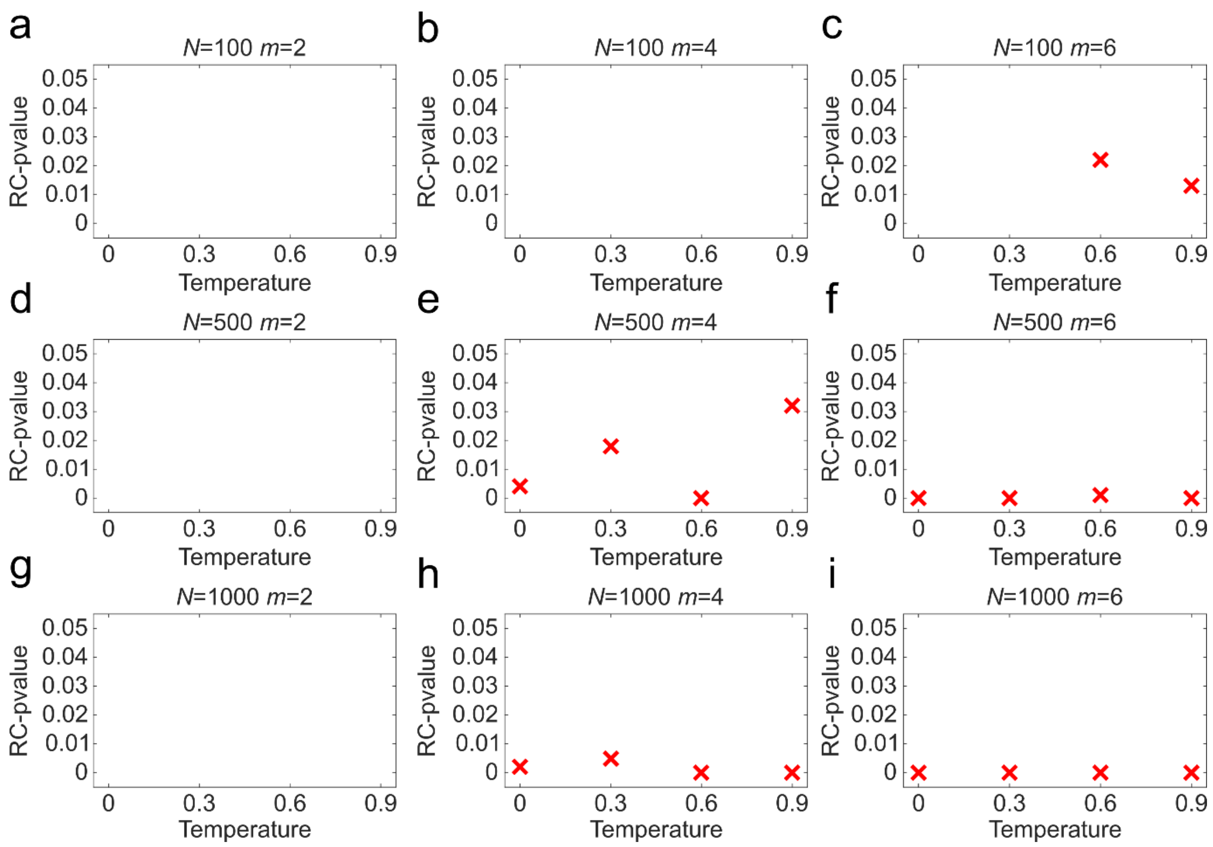

Supplementary Figure 25. Rich-club statistical test on PSO synthetic networks

For each combination of the PSO parameters *N*, *m* and *T*, the statistical test for rich-clubness[1] has been performed on 10 networks and the p-values have been adjusted for multiple hypothesis testing by the Bonferroni correction. For each parameter combination the average of the adjusted p-values is reported, highlighting the range below the significance level of 0.05.

Method	Karate<br>N=34<br>E=78<br>m=2.29<br>T=0.43<br>γ=2.12<br>N_c=2	Opsahl 8<br>N=43<br>E=193<br>m=4.49<br>T=0.43<br>γ=8.20<br>N_c=7	Opsahl 9<br>N=44<br>E=348<br>m=7.91<br>T=0.32<br>γ=5.92<br>N_c=7	Opsahl 10<br>N=77<br>E=518<br>m=6.73<br>T=0.35<br>γ=5.06<br>N_c=4	Opsahl 11<br>N=77<br>E=1088<br>m=14.13<br>T=0.28<br>γ=4.87<br>N_c=4	Polbooks<br>N=105<br>E=441<br>m=4.20<br>T=0.51<br>γ=2.62<br>N_c=3	Football<br>N=115<br>E=613<br>m=5.33<br>T=0.60<br>γ=9.09<br>N_c=12	Polblogs<br>N=1222<br>E=16714<br>m=13.68<br>T=0.68<br>γ=2.38<br>N_c=2	Mean	% Impr.
**EBC-ncISO-EA**	1.00	0.57	0.47	1.00	0.93	0.59	0.90	0.68	0.77	+13.2
**RA-MCE-EA**	0.83	0.51	0.47	1.00	0.96	0.57	0.82	0.67	0.73	+7.4
**RA-ncMCE-EA**	0.73	0.55	0.47	1.00	1.00	0.57	0.83	0.67	0.73	+7.4
**EBC-MCE-EA**	0.83	0.47	0.41	1.00	0.96	0.57	0.90	0.62	0.72	+5.9
**EBC-ncMCE-EA**	0.88	0.46	0.41	1.00	0.96	0.57	0.85	0.62	0.72	+5.9
**EBC-ISO-EA**	0.83	0.42	0.47	1.00	0.89	0.59	0.88	0.66	0.72	+5.9
**LPCS**	0.83	0.49	0.41	1.00	0.96	0.55	0.87	0.67	0.72	+5.9
**ncMCE-EA**	0.73	0.47	0.47	1.00	0.96	0.57	0.89	0.62	0.71	+4.4
**RA-LE-EA**	0.67	0.48	0.53	1.00	0.92	0.56	0.82	0.70	0.71	+4.4
**EBC-ISO**	0.83	0.47	0.47	1.00	0.76	0.54	0.89	0.68	0.71	+4.4
**RA-MCE**	0.83	0.52	0.36	0.93	0.92	0.56	0.85	0.66	0.71	+4.4
**RA-ncISO-EA**	0.67	0.54	0.42	1.00	0.92	0.56	0.86	0.67	0.70	+2.9
**ncISO-EA**	0.73	0.50	0.41	1.00	0.88	0.54	0.87	0.66	0.70	+2.9
**EBC-LE-EA**	0.85	0.42	0.41	0.96	0.92	0.56	0.85	0.62	0.70	+2.9
**RA-ncISO**	0.68	0.46	0.41	1.00	0.87	0.54	0.89	0.72	0.70	+2.9
**EBC-MCE**	0.83	0.42	0.51	1.00	0.92	0.59	0.86	0.40	0.69	+1.5
**EBC-ncISO**	0.68	0.49	0.36	1.00	0.85	0.57	0.85	0.71	0.69	+1.5
**MCE-EA**	0.64	0.47	0.47	0.96	0.92	0.55	0.86	0.62	0.69	+1.5
**RA-ISO**	0.68	0.43	0.39	1.00	0.87	0.54	0.87	0.70	0.69	+1.5
**unweighted**	0.46	0.55	0.41	1.00	0.96	0.50	0.93	0.64	0.68	0.0
**LE**	0.68	0.53	0.37	1.00	0.79	0.53	0.85	0.73	0.68	0.0
**LE-EA**	0.63	0.55	0.41	1.00	0.78	0.55	0.82	0.67	0.68	0.0
**RA-ISO-EA**	0.57	0.43	0.44	1.00	0.88	0.54	0.86	0.67	0.67	-1.5
**ISO**	0.59	0.49	0.45	0.96	0.85	0.56	0.80	0.68	0.67	-1.5
**RA-ncMCE**	0.67	0.45	0.41	0.93	0.89	0.49	0.84	0.64	0.67	-1.5
**RA-LE**	0.55	0.40	0.44	0.96	0.83	0.56	0.85	0.71	0.66	-2.9
**ncISO**	0.68	0.55	0.41	0.96	0.79	0.56	0.68	0.67	0.66	-2.9
**ISO-EA**	0.34	0.50	0.41	0.96	0.93	0.56	0.82	0.67	0.65	-4.4
**HyperMap**	0.56	0.60	0.28	0.92	0.85	0.50	0.83	0.69	0.65	-4.4
**HyperMapCN**	0.55	0.47	0.41	0.93	0.79	0.54	0.79	0.70	0.65	-4.4
**EBC-LE**	0.68	0.36	0.36	1.00	0.79	0.54	0.82	0.60	0.64	-5.9
**EBC-ncMCE**	0.57	0.37	0.49	1.00	0.89	0.56	0.82	0.22	0.62	-8.8
**MCE**	0.73	0.28	0.32	0.50	0.21	0.54	0.77	0.42	0.47	-30.9
**ncMCE**	0.61	0.24	0.25	0.49	0.18	0.49	0.79	0.08	0.39	-42.6

Supplementary Table 1. Community detection on real networks with Louvain algorithm (HSP-kernel)
The table is equivalent to Table 1, but also the non-EA methods are shown.

Method	Karate	Opsahl 8	Opsahl 9	Opsahl 10	Opsahl 11	Polbooks	Football	Polblogs	Mean	% Impr.
	N=34	N=43	N=44	N=77	N=77	N=105	N=115	N=1222		
	E=78	E=193	E=348	E=518	E=1088	E=441	E=613	E=16714		
	m=2.29	m=4.49	m=7.91	m=6.73	m=14.13	m=4.20	m=5.33	m=13.68		
	T=0.43	T=0.43	T=0.32	T=0.35	T=0.28	T=0.51	T=0.60	T=0.68		
	γ=2.12	γ=8.20	γ=5.92	γ=5.06	γ=4.87	γ=2.62	γ=9.09	γ=2.38		
	N_c=2	N_c=7	N_c=7	N_c=4	N_c=4	N_c=3	N_c=12	N_c=2		
**EBC-ncISO-EA**	0.68	0.75	0.47	1.00	1.00	0.54	0.92	0.53	0.74	+4.2
**ncMCE-EA**	0.68	0.74	0.47	1.00	0.93	0.50	0.92	0.52	0.72	+1.4
**unweighted**	0.55	0.69	0.47	1.00	1.00	0.52	0.92	0.52	0.71	0.0
**EBC-MCE-EA**	0.68	0.55	0.53	1.00	0.96	0.52	0.93	0.52	0.71	0.0
**EBC-ncMCE-EA**	0.58	0.55	0.53	1.00	1.00	0.52	0.93	0.52	0.70	-1.4
**ISO-EA**	0.68	0.53	0.47	1.00	0.96	0.52	0.92	0.53	0.70	-1.4
**LE-EA**	0.68	0.54	0.47	1.00	0.96	0.53	0.92	0.51	0.70	-1.4
**EBC-LE-EA**	0.68	0.55	0.47	0.95	0.96	0.52	0.93	0.53	0.70	-1.4
**EBC-ncISO**	0.68	0.52	0.47	1.00	0.93	0.51	0.92	0.54	0.70	-1.4
**RA-ISO**	0.58	0.55	0.47	1.00	1.00	0.52	0.93	0.51	0.70	-1.4
**ncISO-EA**	0.68	0.53	0.47	1.00	0.96	0.47	0.92	0.53	0.69	-2.8
**EBC-ISO-EA**	0.55	0.55	0.47	1.00	1.00	0.52	0.92	0.53	0.69	-2.8
**MCE-EA**	0.68	0.54	0.47	0.95	0.93	0.51	0.92	0.52	0.69	-2.8
**RA-ncISO-EA**	0.55	0.55	0.47	1.00	1.00	0.52	0.92	0.52	0.69	-2.8
**RA-ISO-EA**	0.58	0.55	0.47	1.00	0.96	0.52	0.92	0.52	0.69	-2.8
**RA-ncMCE-EA**	0.47	0.55	0.53	1.00	1.00	0.52	0.92	0.50	0.69	-2.8
**EBC-ISO**	0.57	0.55	0.47	1.00	1.00	0.45	0.92	0.53	0.69	-2.8
**LPCS**	0.55	0.55	0.53	1.00	0.96	0.52	0.93	0.51	0.69	-2.8
**LE**	0.63	0.55	0.36	1.00	1.00	0.48	0.92	0.54	0.68	-4.2
**RA-LE-EA**	0.55	0.55	0.47	1.00	0.93	0.52	0.92	0.52	0.68	-4.2
**EBC-LE**	0.68	0.55	0.36	1.00	0.94	0.47	0.92	0.53	0.68	-4.2
**EBC-ncMCE**	0.54	0.55	0.49	1.00	0.93	0.48	0.93	0.52	0.68	-4.2
**RA-ncISO**	0.54	0.61	0.36	1.00	0.96	0.52	0.92	0.52	0.68	-4.2
**RA-MCE-EA**	0.47	0.55	0.53	1.00	0.92	0.52	0.92	0.51	0.68	-4.2
**ncISO**	0.54	0.53	0.36	1.00	1.00	0.52	0.92	0.52	0.67	-5.6
**ISO**	0.58	0.53	0.36	0.96	0.96	0.52	0.93	0.54	0.67	-5.6
**RA-MCE**	0.47	0.55	0.53	0.93	0.92	0.52	0.92	0.51	0.67	-5.6
**EBC-MCE**	0.58	0.55	0.22	1.00	0.96	0.52	0.93	0.52	0.66	-7.0
**HyperMapCN**	0.52	0.55	0.41	1.00	0.86	0.57	0.89	0.46	0.66	-7.0
**HyperMap**	0.52	0.60	0.32	1.00	0.92	0.49	0.90	0.46	0.65	-8.5
**RA-LE**	0.43	0.55	0.36	1.00	0.96	0.45	0.92	0.53	0.65	-8.5
**RA-ncMCE**	0.47	0.61	0.36	0.93	0.96	0.42	0.92	0.44	0.64	-9.9
**MCE**	0.57	0.53	0.55	0.93	0.50	0.59	0.92	0.38	0.62	-12.7
**ncMCE**	0.54	0.45	0.59	0.98	0.50	0.41	0.92	0.38	0.60	-15.5

Supplementary Table 2. Community detection on real networks with Infomap algorithm
The table is equivalent to Table 2, but also the non-EA methods are shown.

Method	Karate<br>N=34<br>E=78<br>m=2.29<br>T=0.43<br>γ=2.12<br>N_c=2	Opsahl 8<br>N=43<br>E=193<br>m=4.49<br>T=0.43<br>γ=8.20<br>N_c=7	Opsahl 9<br>N=44<br>E=348<br>m=7.91<br>T=0.32<br>γ=5.92<br>N_c=7	Opsahl 10<br>N=77<br>E=518<br>m=6.73<br>T=0.35<br>γ=5.06<br>N_c=4	Opsahl 11<br>N=77<br>E=1088<br>m=14.13<br>T=0.28<br>γ=4.87<br>N_c=4	Polbooks<br>N=105<br>E=441<br>m=4.20<br>T=0.51<br>γ=2.62<br>N_c=3	Football<br>N=115<br>E=613<br>m=5.33<br>T=0.60<br>γ=9.09<br>N_c=12	Polblogs<br>N=1222<br>E=16714<br>m=13.68<br>T=0.68<br>γ=2.38<br>N_c=2	Mean	% Impr.
**ISO**	0.63	0.50	0.25	0.94	0.83	0.56	0.91	0.69	0.67	+9.8
**EBC-ncISO-EA**	0.72	0.53	0.42	0.96	0.43	0.52	0.91	0.68	0.65	+6.6
**ncISO-EA**	0.75	0.38	0.50	0.95	0.44	0.52	0.89	0.69	0.64	+4.9
**EBC-ncISO**	0.52	0.58	0.32	1.00	0.57	0.51	0.91	0.68	0.64	+4.9
**RA-ISO**	0.58	0.46	0.42	1.00	0.54	0.50	0.90	0.68	0.63	+3.3
**RA-ncMCE-EA**	0.68	0.51	0.53	0.97	0.29	0.54	0.90	0.64	0.63	+3.3
**EBC-ISO**	0.50	0.40	0.42	0.99	0.66	0.51	0.90	0.66	0.63	+3.3
**EBC-ncMCE**	0.42	0.47	0.40	0.97	0.70	0.49	0.91	0.69	0.63	+3.3
**RA-MCE**	0.62	0.41	0.38	0.99	0.71	0.51	0.87	0.55	0.63	+3.3
**RA-LE**	0.43	0.40	0.28	1.00	0.83	0.49	0.91	0.68	0.63	+3.3
**RA-ncISO**	0.44	0.51	0.24	1.00	0.73	0.52	0.89	0.69	0.63	+3.3
**LE**	0.63	0.49	0.27	1.00	0.48	0.51	0.91	0.70	0.62	+1.6
**EBC-LE-EA**	0.76	0.46	0.34	0.92	0.38	0.54	0.91	0.68	0.62	+1.6
**LPCS**	0.49	0.48	0.29	1.00	0.60	0.53	0.89	0.68	0.62	+1.6
**unweighted**	0.73	0.44	0.27	0.99	0.34	0.56	0.88	0.70	0.61	0.0
**HyperMap**	0.52	0.45	0.25	0.98	0.63	0.50	0.86	0.66	0.61	0.0
**EBC-MCE**	0.54	0.47	0.33	1.00	0.42	0.54	0.90	0.62	0.60	-1.6
**RA-ncISO-EA**	0.60	0.48	0.30	0.98	0.33	0.56	0.89	0.68	0.60	-1.6
**RA-ISO-EA**	0.79	0.44	0.14	0.96	0.35	0.53	0.89	0.69	0.60	-1.6
**EBC-MCE-EA**	0.82	0.44	0.19	0.95	0.29	0.56	0.91	0.61	0.60	-1.6
**ISO-EA**	0.57	0.43	0.19	0.99	0.44	0.56	0.90	0.69	0.60	-1.6
**EBC-LE**	0.50	0.45	0.29	1.00	0.58	0.51	0.89	0.54	0.60	-1.6
**ncISO**	0.50	0.41	0.20	0.94	0.60	0.50	0.89	0.70	0.59	-3.3
**LE-EA**	0.70	0.57	0.11	0.92	0.33	0.54	0.90	0.68	0.59	-3.3
**EBC-ncMCE-EA**	0.67	0.41	0.29	0.90	0.36	0.53	0.90	0.68	0.59	-3.3
**RA-ncMCE**	0.45	0.53	0.32	0.86	0.72	0.37	0.85	0.63	0.59	-3.3
**HyperMapCN**	0.48	0.46	0.38	0.87	0.61	0.42	0.82	0.67	0.59	-3.3
**EBC-ISO-EA**	0.66	0.38	0.33	0.95	0.29	0.53	0.90	0.68	0.59	-3.3
**RA-LE-EA**	0.62	0.53	0.26	0.96	0.22	0.52	0.89	0.68	0.59	-3.3
**RA-MCE-EA**	0.39	0.43	0.37	0.94	0.28	0.55	0.89	0.70	0.57	-6.6
**ncMCE-EA**	0.67	0.42	0.17	0.96	0.14	0.56	0.88	0.69	0.56	-8.2
**ncMCE**	0.58	0.59	0.23	0.78	0.32	0.42	0.87	0.62	0.55	-9.8
**MCE-EA**	0.56	0.24	0.29	0.92	0.19	0.56	0.88	0.71	0.55	-9.8
**MCE**	0.54	0.47	0.37	0.63	0.30	0.44	0.87	0.63	0.53	-13.1

Supplementary Table 3. Community detection on real networks with Label propagation algorithm
The table is equivalent to Supplementary Table 2, but the Label propagation algorithm is used rather than Infomap.

Method	Karate<br>N=34<br>E=78<br>m=2.29<br>T=0.43<br>γ=2.12<br>N_c=2	Opsahl 8<br>N=43<br>E=193<br>m=4.49<br>T=0.43<br>γ=8.20<br>N_c=7	Opsahl 9<br>N=44<br>E=348<br>m=7.91<br>T=0.32<br>γ=5.92<br>N_c=7	Opsahl 10<br>N=77<br>E=518<br>m=6.73<br>T=0.35<br>γ=5.06<br>N_c=4	Opsahl 11<br>N=77<br>E=1088<br>m=14.13<br>T=0.28<br>γ=4.87<br>N_c=4	Polbooks<br>N=105<br>E=441<br>m=4.20<br>T=0.51<br>γ=2.62<br>N_c=3	Football<br>N=115<br>E=613<br>m=5.33<br>T=0.60<br>γ=9.09<br>N_c=12	Polblogs<br>N=1222<br>E=16714<br>m=13.68<br>T=0.68<br>γ=2.38<br>N_c=2	Mean	% Impr.
**RA-ISO-EA**	0.58	0.44	0.53	1.00	0.85	0.56	0.89	0.65	0.69	+6.2
**RA-MCE-EA**	0.49	0.41	0.53	1.00	0.96	0.54	0.91	0.65	0.69	+6.2
**ncISO-EA**	0.68	0.44	0.47	1.00	0.89	0.54	0.86	0.67	0.69	+6.2
**LPCS**	0.57	0.55	0.41	1.00	0.96	0.54	0.89	0.63	0.69	+6.2
**EBC-ncISO-EA**	0.68	0.47	0.36	1.00	0.89	0.54	0.89	0.65	0.68	+4.6
**EBC-MCE-EA**	0.68	0.44	0.36	1.00	0.91	0.54	0.89	0.65	0.68	+4.6
**EBC-ncMCE-EA**	0.68	0.44	0.36	1.00	0.89	0.54	0.89	0.64	0.68	+4.6
**ncMCE-EA**	0.58	0.41	0.47	1.00	0.89	0.54	0.89	0.67	0.68	+4.6
**EBC-MCE**	0.49	0.51	0.36	1.00	0.96	0.54	0.89	0.67	0.68	+4.6
**RA-ncMCE**	0.53	0.55	0.46	0.91	1.00	0.48	0.89	0.61	0.68	+4.6
**RA-LE-EA**	0.58	0.53	0.36	1.00	0.90	0.54	0.91	0.64	0.68	+4.6
**RA-ncMCE-EA**	0.51	0.44	0.41	1.00	0.96	0.54	0.89	0.66	0.68	+4.6
**EBC-ISO-EA**	0.47	0.45	0.47	1.00	0.89	0.54	0.89	0.66	0.67	+3.1
**EBC-LE-EA**	0.68	0.44	0.36	0.90	0.89	0.54	0.86	0.66	0.67	+3.1
**HyperMapCN**	0.55	0.55	0.41	1.00	0.83	0.46	0.94	0.61	0.67	+3.1
**MCE-EA**	0.52	0.41	0.47	0.92	0.89	0.54	0.89	0.68	0.66	+1.5
**EBC-ncISO**	0.68	0.53	0.36	1.00	0.83	0.44	0.85	0.62	0.66	+1.5
**LE**	0.68	0.44	0.36	1.00	0.86	0.45	0.89	0.61	0.66	+1.5
**unweighted**	0.46	0.45	0.36	1.00	0.89	0.53	0.89	0.64	0.65	0.0
**RA-ISO**	0.68	0.44	0.36	1.00	0.73	0.54	0.85	0.63	0.65	0.0
**ISO-EA**	0.46	0.44	0.36	1.00	0.89	0.54	0.86	0.67	0.65	0.0
**LE-EA**	0.68	0.44	0.36	1.00	0.73	0.54	0.85	0.62	0.65	0.0
**RA-ncISO-EA**	0.50	0.44	0.36	1.00	0.85	0.54	0.85	0.66	0.65	0.0
**ISO**	0.52	0.44	0.36	0.96	0.86	0.54	0.86	0.63	0.65	0.0
**RA-MCE**	0.50	0.48	0.41	0.91	0.87	0.50	0.85	0.65	0.65	0.0
**EBC-ISO**	0.57	0.41	0.47	1.00	0.80	0.44	0.89	0.60	0.65	0.0
**EBC-ncMCE**	0.47	0.35	0.42	1.00	0.90	0.46	0.91	0.64	0.64	-1.5
**ncISO**	0.48	0.44	0.36	1.00	0.89	0.49	0.86	0.63	0.64	-1.5
**RA-ncISO**	0.50	0.48	0.36	1.00	0.76	0.54	0.85	0.62	0.64	-1.5
**RA-LE**	0.51	0.35	0.36	1.00	0.87	0.44	0.91	0.64	0.63	-3.1
**EBC-LE**	0.57	0.35	0.36	1.00	0.80	0.45	0.89	0.65	0.63	-3.1
**HyperMap**	0.45	0.56	0.36	0.96	0.78	0.43	0.87	0.62	0.63	-3.1
**ncMCE**	0.46	0.52	0.47	0.92	0.55	0.51	0.89	0.33	0.58	-10.8
**MCE**	0.42	0.42	0.47	0.92	0.56	0.47	0.91	0.33	0.56	-13.8

Supplementary Table 4. Community detection on real networks with Walktrap algorithm
The table is equivalent to Supplementary Table 2, but the Walktrap algorithm is used rather than Infomap.

Method	Karate	Opsahl 8	Opsahl 9	Opsahl 10	Opsahl 11	Polbooks	Football	Polblogs	Mean	% Impr.
	N=34	N=43	N=44	N=77	N=77	N=105	N=115	N=1222		
	E=78	E=193	E=348	E=518	E=1088	E=441	E=613	E=16714		
	m=2.29	m=4.49	m=7.91	m=6.73	m=14.13	m=4.20	m=5.33	m=13.68		
	T=0.43	T=0.43	T=0.32	T=0.35	T=0.28	T=0.51	T=0.60	T=0.68		
	γ=2.12	γ=8.20	γ=5.92	γ=5.06	γ=4.87	γ=2.62	γ=9.09	γ=2.38		
	N_c=2	N_c=7	N_c=7	N_c=4	N_c=4	N_c=3	N_c=12	N_c=2		
**EBC-ISO-EA**	0.78	0.50	0.41	1.00	0.96	0.45	0.90	0.63	0.70	+2.9
**ISO-EA**	0.58	0.50	0.46	1.00	1.00	0.51	0.93	0.66	0.70	+2.9
**MCE-EA**	0.51	0.55	0.53	0.96	0.96	0.54	0.90	0.66	0.70	+2.9
**RA-ISO**	0.68	0.55	0.39	1.00	0.96	0.49	0.90	0.62	0.70	+2.9
**EBC-ncMCE**	0.57	0.55	0.49	1.00	0.96	0.49	0.91	0.60	0.70	+2.9
**EBC-ncISO-EA**	0.56	0.55	0.41	1.00	0.93	0.52	0.90	0.62	0.69	+1.5
**EBC-ncMCE-EA**	0.47	0.55	0.41	1.00	0.96	0.54	0.93	0.62	0.69	+1.5
**EBC-MCE-EA**	0.57	0.55	0.41	1.00	0.96	0.51	0.91	0.62	0.69	+1.5
**EBC-ISO**	0.57	0.50	0.47	1.00	1.00	0.45	0.93	0.64	0.69	+1.5
**EBC-ncISO**	0.68	0.50	0.37	1.00	0.96	0.45	0.90	0.67	0.69	+1.5
**EBC-LE-EA**	0.68	0.51	0.42	0.96	0.92	0.52	0.87	0.63	0.69	+1.5
**LE-EA**	0.59	0.55	0.42	1.00	0.93	0.51	0.90	0.63	0.69	+1.5
**LE**	0.58	0.55	0.41	1.00	0.96	0.50	0.90	0.65	0.69	+1.5
**EBC-LE**	0.68	0.50	0.41	1.00	0.96	0.45	0.90	0.62	0.69	+1.5
**unweighted**	0.46	0.55	0.41	1.00	0.96	0.50	0.93	0.64	0.68	0.0
**RA-ncISO-EA**	0.49	0.55	0.41	1.00	0.96	0.52	0.90	0.63	0.68	0.0
**ncISO-EA**	0.52	0.46	0.46	1.00	0.96	0.48	0.91	0.66	0.68	0.0
**EBC-MCE**	0.47	0.49	0.49	1.00	0.96	0.54	0.90	0.59	0.68	0.0
**RA-LE-EA**	0.48	0.55	0.46	1.00	0.96	0.46	0.90	0.63	0.68	0.0
**RA-ISO-EA**	0.50	0.50	0.41	1.00	0.96	0.52	0.90	0.64	0.68	0.0
**ISO**	0.47	0.50	0.41	0.96	0.96	0.56	0.91	0.66	0.68	0.0
**ncISO**	0.47	0.52	0.42	1.00	0.96	0.47	0.91	0.67	0.68	0.0
**RA-MCE-EA**	0.49	0.52	0.42	1.00	0.92	0.52	0.91	0.64	0.68	0.0
**RA-ncMCE-EA**	0.47	0.55	0.42	1.00	0.96	0.46	0.91	0.64	0.68	0.0
**ncMCE-EA**	0.50	0.48	0.41	1.00	0.96	0.50	0.90	0.66	0.68	0.0
**RA-LE**	0.55	0.48	0.41	1.00	0.92	0.45	0.89	0.65	0.67	-1.5
**RA-ncISO**	0.47	0.48	0.41	1.00	0.92	0.47	0.90	0.63	0.66	-2.9
**RA-MCE**	0.47	0.54	0.42	0.93	0.92	0.51	0.86	0.63	0.66	-2.9
**RA-ncMCE**	0.47	0.60	0.41	0.93	1.00	0.46	0.88	0.48	0.66	-2.9
**LPCS**	0.47	0.50	0.41	1.00	0.96	0.46	0.89	0.61	0.66	-2.9
**HyperMapCN**	0.55	0.49	0.41	1.00	0.88	0.46	0.90	0.60	0.66	-2.9
**HyperMap**	0.56	0.57	0.32	1.00	0.92	0.47	0.87	0.57	0.66	-2.9
**MCE**	0.52	0.46	0.34	0.75	0.56	0.47	0.88	0.54	0.56	-17.6
**ncMCE**	0.54	0.49	0.34	0.82	0.40	0.43	0.89	0.55	0.56	-17.6

Supplementary Table 5. Community detection on real networks with Louvain algorithm (HD-network) The table is equivalent to Supplementary Table 1, but the HD-weighted network (see Equation 13) is given in input to the Louvain algorithm rather than the HSP-kernel (see Equation 14).

Method	AS 201501 IPv6	AS 200909 IPv4	AS 200912 IPv4	AS 201003 IPv4	AS 201006 IPv4	AS 201009 IPv4	AS 201012 IPv4	AS 201501 IPv4	Mean	% Impr.
	N=5143	N=24091	N=25910	N=26307	N=26756	N=28353	N=29333	N=37542		
	E=13446	E=59531	E=63435	E=66089	E=68150	E=73722	E=78054	E=95019		
	m=2.61	m=2.47	m=2.45	m=2.51	m=2.55	m=2.60	m=2.66	m=2.53		
	T=0.65	T=0.64	T=0.64	T=0.63	T=0.63	T=0.63	T=0.62	T=0.64		
	γ=2.30	γ=2.12	γ=2.11	γ=2.26	γ=2.08	γ=2.23	γ=2.22	γ=2.07		
	N_c=151	N_c=203	N_c=206	N_c=204	N_c=204	N_c=208	N_c=212	N_c=222		
**EBC-ncISO-EA**	0.54	0.61	0.59	0.60	0.60	0.62	0.59	0.62	0.60	+3.4
**EBC-ISO-EA**	0.53	0.61	0.60	0.59	0.59	0.62	0.61	0.62	0.60	+3.4
**EBC-ISO**	0.52	0.61	0.60	0.61	0.61	0.62	0.62	0.62	0.60	+3.4
**EBC-ncISO**	0.53	0.61	0.60	0.60	0.59	0.62	0.62	0.60	0.60	+3.4
**EBC-MCE**	0.54	0.61	0.60	0.61	0.61	0.60	0.60	0.62	0.60	+3.4
**EBC-ncMCE-EA**	0.54	0.61	0.59	0.60	0.60	0.61	0.60	0.59	0.59	+1.7
**RA-ncMCE-EA**	0.56	0.60	0.59	0.59	0.59	0.59	0.59	0.59	0.59	+1.7
**EBC-MCE-EA**	0.54	0.60	0.59	0.61	0.61	0.60	0.60	0.58	0.59	+1.7
**RA-MCE-EA**	0.57	0.60	0.59	0.59	0.58	0.59	0.59	0.59	0.59	+1.7
**EBC-LE-EA**	0.55	0.58	0.60	0.60	0.59	0.60	0.60	0.58	0.59	+1.7
**RA-LE-EA**	0.54	0.61	0.58	0.59	0.60	0.60	0.59	0.59	0.59	+1.7
**EBC-ncMCE**	0.53	0.61	0.59	0.60	0.61	0.59	0.60	0.61	0.59	+1.7
**RA-ncMCE**	0.57	0.60	0.59	0.59	0.59	0.59	0.59	0.59	0.59	+1.7
**RA-MCE**	0.56	0.59	0.59	0.59	0.59	0.59	0.59	0.59	0.59	+1.7
**MCE-EA**	0.55	0.60	0.59	0.59	0.60	0.59	0.59	0.59	0.59	+1.7
**unweighted**	0.56	0.58	0.58	0.58	0.58	0.59	0.59	0.58	0.58	0.0
**RA-ISO-EA**	0.55	0.59	0.59	0.59	0.59	0.59	0.59	0.59	0.58	0.0
**RA-ncISO**	0.55	0.60	0.58	0.58	0.59	0.59	0.60	0.58	0.58	0.0
**EBC-LE**	0.55	0.57	0.59	0.60	0.59	0.62	0.57	0.57	0.58	0.0
**RA-LE**	0.55	0.57	0.58	0.59	0.60	0.62	0.57	0.59	0.58	0.0
**RA-ISO**	0.55	0.59	0.58	0.59	0.59	0.59	0.59	0.59	0.58	0.0
**RA-ncISO-EA**	0.55	0.59	0.58	0.59	0.58	0.59	0.59	0.58	0.58	0.0
**LE-EA**	0.55	0.60	0.58	0.58	0.58	0.59	0.58	0.58	0.58	0.0
**ncMCE-EA**	0.55	0.59	0.58	0.59	0.58	0.59	0.59	0.58	0.58	0.0
**MCE**	0.55	0.59	0.58	0.58	0.58	0.59	0.59	0.58	0.58	0.0
**ncISO**	0.53	0.59	0.58	0.58	0.58	0.60	0.60	0.58	0.58	0.0
**ncMCE**	0.55	0.59	0.58	0.57	0.58	0.59	0.58	0.59	0.58	0.0
**ncISO-EA**	0.53	0.59	0.58	0.58	0.58	0.60	0.59	0.58	0.58	0.0
**LE**	0.55	0.58	0.58	0.59	0.58	0.58	0.58	0.59	0.58	0.0
**ISO**	0.52	0.59	0.58	0.58	0.58	0.58	0.59	0.58	0.57	-1.7
**ISO-EA**	0.53	0.58	0.57	0.58	0.58	0.58	0.59	0.57	0.57	-1.7
**LPCS**	0.56	0.57	0.57	0.58	0.58	0.57	0.58	0.57	0.57	-1.7

Supplementary Table 6. Community detection on Internet networks with Infomap algorithm

The table is equivalent to Supplementary Table 2, but the community detection is performed on the Internet networks rather than on the small-size real networks.

Method	AS 201501 IPv6	AS 200909 IPv4	AS 200912 IPv4	AS 201003 IPv4	AS 201006 IPv4	AS 201009 IPv4	AS 201012 IPv4	AS 201501 IPv4	Mean	% Impr.
	N=5143	N=24091	N=25910	N=26307	N=26756	N=28353	N=29333	N=37542		
	E=13446	E=59531	E=63435	E=66089	E=68150	E=73722	E=78054	E=95019		
	m=2.61	m=2.47	m=2.45	m=2.51	m=2.55	m=2.60	m=2.66	m=2.53		
	T=0.65	T=0.64	T=0.64	T=0.63	T=0.63	T=0.63	T=0.62	T=0.64		
	γ=2.30	γ=2.12	γ=2.11	γ=2.26	γ=2.08	γ=2.23	γ=2.22	γ=2.07		
	N_c=151	N_c=203	N_c=206	N_c=204	N_c=204	N_c=208	N_c=212	N_c=222		
**LPCS**	0.51	0.55	0.51	0.56	0.52	0.54	0.46	0.32	0.50	+117.4
**ncMCE**	0.32	0.44	0.36	0.55	0.28	0.28	0.43	0.21	0.36	+56.5
**RA-MCE**	0.21	0.26	0.50	0.36	0.29	0.47	0.24	0.32	0.33	+43.5
**EBC-LE**	0.36	0.44	0.35	0.26	0.19	0.28	0.37	0.34	0.33	+43.5
**LE**	0.15	0.40	0.43	0.40	0.34	0.33	0.26	0.27	0.32	+39.1
**RA-ncISO**	0.30	0.37	0.37	0.42	0.27	0.32	0.27	0.18	0.31	+34.8
**MCE**	0.27	0.38	0.35	0.39	0.27	0.26	0.26	0.24	0.30	+30.4
**RA-ncMCE**	0.20	0.33	0.30	0.29	0.51	0.22	0.39	0.19	0.30	+30.4
**LE-EA**	0.14	0.40	0.39	0.32	0.33	0.28	0.27	0.23	0.29	+26.1
**RA-LE**	0.35	0.44	0.27	0.27	0.17	0.31	0.38	0.17	0.29	+26.1
**ncMCE-EA**	0.32	0.35	0.31	0.36	0.23	0.23	0.30	0.21	0.29	+26.1
**MCE-EA**	0.19	0.35	0.31	0.34	0.26	0.24	0.24	0.18	0.27	+17.4
**RA-ISO**	0.12	0.29	0.36	0.27	0.25	0.32	0.23	0.16	0.25	+8.7
**EBC-LE-EA**	0.13	0.39	0.28	0.28	0.21	0.23	0.22	0.20	0.24	+4.3
**RA-MCE-EA**	0.22	0.29	0.32	0.26	0.22	0.23	0.23	0.17	0.24	+4.3
**unweighted**	0.07	0.30	0.41	0.29	0.29	0.19	0.19	0.15	0.23	0.0
**RA-ncMCE-EA**	0.16	0.23	0.25	0.25	0.36	0.22	0.28	0.14	0.23	0.0
**EBC-ISO-EA**	0.15	0.23	0.32	0.25	0.21	0.27	0.27	0.18	0.23	0.0
**ncISO**	0.08	0.24	0.31	0.29	0.26	0.27	0.19	0.17	0.23	0.0
**EBC-ncMCE-EA**	0.10	0.24	0.25	0.27	0.22	0.24	0.22	0.19	0.22	-4.3
**EBC-ncISO-EA**	0.12	0.23	0.26	0.25	0.23	0.25	0.24	0.19	0.22	-4.3
**EBC-ncISO**	0.18	0.22	0.22	0.25	0.25	0.22	0.22	0.19	0.22	-4.3
**RA-LE-EA**	0.13	0.29	0.24	0.24	0.19	0.20	0.20	0.19	0.21	-8.7
**ISO**	0.09	0.30	0.29	0.25	0.18	0.21	0.19	0.16	0.21	-8.7
**EBC-ISO**	0.12	0.29	0.24	0.22	0.20	0.20	0.20	0.19	0.21	-8.7
**ISO-EA**	0.10	0.30	0.27	0.26	0.17	0.22	0.19	0.15	0.21	-8.7
**EBC-MCE-EA**	0.10	0.21	0.23	0.24	0.19	0.26	0.20	0.16	0.20	-13.0
**RA-ncISO-EA**	0.13	0.30	0.24	0.27	0.14	0.20	0.17	0.16	0.20	-13.0
**ncISO-EA**	0.08	0.21	0.36	0.26	0.21	0.21	0.17	0.13	0.20	-13.0
**RA-ISO-EA**	0.11	0.21	0.29	0.25	0.23	0.24	0.18	0.14	0.20	-13.0
**EBC-MCE**	0.07	0.21	0.26	0.22	0.22	0.25	0.19	0.16	0.20	-13.0
**EBC-ncMCE**	0.08	0.24	0.25	0.21	0.23	0.19	0.16	0.19	0.19	-17.4

Supplementary Table 7. Community detection on Internet networks with Label propagation algorithm
The table is equivalent to Supplementary Table 6, but the Label propagation algorithm is used rather than Infomap.

Method	AS 201501 IPv6	AS 200909 IPv4	AS 200912 IPv4	AS 201003 IPv4	AS 201006 IPv4	AS 201009 IPv4	AS 201012 IPv4	AS 201501 IPv4	Mean	% Impr.
	N=5143	N=24091	N=25910	N=26307	N=26756	N=28353	N=29333	N=37542		
	E=13446	E=59531	E=63435	E=66089	E=68150	E=73722	E=78054	E=95019		
	m=2.61	m=2.47	m=2.45	m=2.51	m=2.55	m=2.60	m=2.66	m=2.53		
	T=0.65	T=0.64	T=0.64	T=0.63	T=0.63	T=0.63	T=0.62	T=0.64		
	γ=2.30	γ=2.12	γ=2.11	γ=2.26	γ=2.08	γ=2.23	γ=2.22	γ=2.07		
	N_c=151	N_c=203	N_c=206	N_c=204	N_c=204	N_c=208	N_c=212	N_c=222		
**RA-MCE**	0.52	0.62	0.66	0.63	0.64	0.65	0.64	0.64	0.63	+1.6
**unweighted**	0.51	0.65	0.64	0.63	0.63	0.64	0.65	0.63	0.62	0.0
**RA-MCE-EA**	0.52	0.63	0.65	0.62	0.63	0.65	0.65	0.64	0.62	0.0
**RA-ncISO-EA**	0.51	0.63	0.65	0.64	0.63	0.66	0.64	0.63	0.62	0.0
**EBC-ncISO-EA**	0.51	0.65	0.64	0.62	0.64	0.64	0.65	0.63	0.62	0.0
**EBC-MCE-EA**	0.48	0.63	0.65	0.62	0.63	0.65	0.64	0.61	0.62	0.0
**RA-ncMCE-EA**	0.51	0.64	0.65	0.64	0.60	0.64	0.65	0.63	0.62	0.0
**EBC-ISO-EA**	0.48	0.63	0.61	0.64	0.65	0.65	0.65	0.61	0.62	0.0
**RA-ISO-EA**	0.49	0.64	0.65	0.65	0.61	0.64	0.64	0.63	0.62	0.0
**EBC-ncISO**	0.48	0.64	0.65	0.65	0.64	0.65	0.65	0.61	0.62	0.0
**RA-ncMCE**	0.51	0.63	0.64	0.61	0.65	0.64	0.64	0.65	0.62	0.0
**MCE**	0.51	0.64	0.64	0.65	0.63	0.63	0.64	0.62	0.62	0.0
**EBC-ISO**	0.46	0.64	0.65	0.64	0.64	0.66	0.65	0.62	0.62	0.0
**LE-EA**	0.49	0.64	0.63	0.65	0.62	0.65	0.64	0.64	0.62	0.0
**ISO**	0.48	0.65	0.64	0.64	0.63	0.64	0.64	0.62	0.62	0.0
**ncISO-EA**	0.49	0.64	0.64	0.64	0.63	0.61	0.64	0.64	0.62	0.0
**LE**	0.50	0.67	0.64	0.62	0.62	0.65	0.64	0.62	0.62	0.0
**MCE-EA**	0.51	0.63	0.63	0.64	0.63	0.64	0.65	0.62	0.62	0.0
**ncISO**	0.47	0.63	0.64	0.64	0.63	0.63	0.64	0.64	0.62	0.0
**LPCS**	0.53	0.63	0.64	0.63	0.63	0.65	0.62	0.63	0.62	0.0
**EBC-ncMCE-EA**	0.47	0.61	0.65	0.63	0.63	0.65	0.64	0.62	0.61	-1.6
**ncMCE-EA**	0.51	0.63	0.63	0.64	0.63	0.64	0.61	0.63	0.61	-1.6
**ISO-EA**	0.49	0.64	0.64	0.63	0.62	0.64	0.64	0.62	0.61	-1.6
**EBC-LE-EA**	0.49	0.63	0.63	0.63	0.62	0.64	0.64	0.61	0.61	-1.6
**RA-LE-EA**	0.48	0.63	0.64	0.63	0.62	0.64	0.63	0.62	0.61	-1.6
**EBC-LE**	0.53	0.62	0.64	0.62	0.63	0.64	0.61	0.61	0.61	-1.6
**RA-LE**	0.53	0.61	0.62	0.64	0.62	0.63	0.61	0.62	0.61	-1.6
**EBC-MCE**	0.45	0.62	0.65	0.61	0.62	0.65	0.64	0.63	0.61	-1.6
**EBC-ncMCE**	0.44	0.65	0.65	0.61	0.61	0.64	0.64	0.62	0.61	-1.6
**RA-ISO**	0.48	0.60	0.61	0.65	0.62	0.65	0.62	0.62	0.61	-1.6
**RA-ncISO**	0.52	0.60	0.63	0.62	0.63	0.63	0.63	0.60	0.61	-1.6
**ncMCE**	0.51	0.62	0.62	0.61	0.62	0.64	0.61	0.63	0.61	-1.6

Supplementary Table 8. Community detection on Internet networks with Walktrap algorithm
The table is equivalent to Supplementary Table 6, but the Walktrap algorithm is used rather than Infomap.

Method	Mean GR-score 2D	Mean GR-score 3D	Improvement	p-value
**ISO**	0.61	0.67	+0.06	**< 0.001**
**ncISO**	0.62	0.66	+0.04	**< 0.001**
**RA-ISO**	0.64	0.65	+0.01	0.296
**EBC-ISO**	0.61	0.61	0.00	0.494
**EBC-ncISO**	0.62	0.62	0.00	0.499
**RA-ncISO**	0.65	0.65	0.00	0.429
**LE**	0.67	0.66	-0.01	0.296
**RA-LE**	0.68	0.67	-0.01	0.259
**EBC-LE**	0.68	0.66	-0.02	0.113

Supplementary Table 9. Comparison of 2D and 3D greedy routing on PSO synthetic networks
The same PSO networks considered in Fig. 3 have been mapped both in 2D and 3D using the manifold-based coalescent embedding techniques and the greedy routing in the hyperbolic space has been evaluated. The table reports for each method the mean GR-score over all the PSO parameter combinations, both in 2D and in 3D, highlighting the 3D-improvement. The GR-score is a metric to evaluate the efficiency of the greedy routing, which assumes values between 0, when all the routings are unsuccessful, and 1, when all the packets reach the destination through the shortest path (see Methods for details). The rightmost column shows for each method the p-value of the permutation test for the mean (10000 iterations) performed considering the two vectors of GR-scores related to 2D and 3D. The p-values lower than the significance level of 0.05 are highlighted in bold.

GR-score 2D

Method	Karate	Opsahl 8	Opsahl 9	Opsahl 10	Opsahl 11	Polbooks	Football	Polblogs	Mean
RA-ncISO	0.91	0.92	0.95	0.92	0.96	0.47	0.65	0.41	0.77
RA-LE	0.85	0.91	0.97	0.91	0.98	0.49	0.70	0.30	0.76
RA-ISO	0.86	0.93	0.93	0.90	0.96	0.43	0.62	0.43	0.76
ncISO	0.82	0.94	0.95	0.91	0.97	0.40	0.60	0.44	0.75
ISO	0.79	0.93	0.91	0.88	0.98	0.40	0.56	0.51	0.75
LE	0.80	0.93	0.97	0.93	0.94	0.42	0.59	0.38	0.74
EBC-LE	0.78	0.92	0.95	0.93	0.91	0.40	0.72	0.26	0.73
EBC-ncISO	0.80	0.83	0.94	0.91	0.92	0.40	0.58	0.45	0.73
EBC-ISO	0.84	0.89	0.94	0.85	0.94	0.36	0.57	0.38	0.72

GR-score 3D

Method	Karate	Opsahl 8	Opsahl 9	Opsahl 10	Opsahl 11	Polbooks	Football	Polblogs	Mean
RA-ISO	0.86	0.97	0.97	0.96	0.94	0.44	0.77	0.47	0.80
RA-ncISO	0.80	0.96	0.97	0.96	0.94	0.44	0.76	0.46	0.79
ncISO	0.76	0.97	0.97	0.97	0.94	0.44	0.67	0.49	0.78
LE	0.75	0.91	0.98	0.94	0.94	0.47	0.75	0.46	0.77
RA-LE	0.75	0.89	0.98	0.96	0.94	0.51	0.75	0.38	0.77
EBC-ISO	0.80	0.92	0.95	0.93	0.92	0.41	0.76	0.46	0.77
EBC-ncISO	0.76	0.93	0.96	0.92	0.92	0.41	0.78	0.45	0.77
ISO	0.72	0.94	0.97	0.96	0.94	0.44	0.65	0.51	0.77
EBC-LE	0.79	0.88	0.92	0.95	0.93	0.43	0.74	0.11	0.72

Improvement

Method	Karate	Opsahl 8	Opsahl 9	Opsahl 10	Opsahl 11	Polbooks	Football	Polblogs	Mean	p-value
EBC-ISO	-0.04	0.03	0.01	0.08	-0.02	0.05	0.19	0.08	+0.05	0.409
EBC-ncISO	-0.04	0.10	0.02	0.01	0.00	0.01	0.20	0.00	+0.04	0.412
RA-ISO	0.00	0.04	0.04	0.06	-0.02	0.01	0.15	0.04	+0.04	0.359
LE	-0.05	-0.02	0.01	0.01	0.00	0.05	0.16	0.08	+0.03	0.376
ncISO	-0.06	0.03	0.02	0.06	-0.03	0.04	0.07	0.05	+0.03	0.434
ISO	-0.07	0.01	0.06	0.08	-0.04	0.04	0.09	0.00	+0.02	0.424
RA-ncISO	-0.11	0.04	0.02	0.04	-0.02	-0.03	0.11	0.05	+0.02	0.451
RA-LE	-0.10	-0.02	0.01	0.05	-0.04	0.02	0.05	0.08	+0.01	0.421
EBC-LE	0.01	-0.04	-0.03	0.02	0.02	0.03	0.02	-0.15	-0.01	0.493

Supplementary Table 10. Comparison of 2D and 3D greedy routing on real networks
The 8 real networks whose statistics are reported in Table 1 have been mapped both in 2D and 3D using the manifold-based coalescent embedding techniques and the greedy routing in the hyperbolic space has been evaluated. The table reports for each method and for each network the GR-score both in 2D and in 3D, in addition to the 3D-improvement. The GR-score is a metric to evaluate the efficiency of the greedy routing, which assumes values between 0, when all the routings are unsuccessful, and 1, when all the packets reach the destination through the shortest path (see Methods for details). The rightmost column shows for each method the p-value of the permutation test for the mean (10000 iterations) performed considering the two vectors of GR-scores related to 2D and 3D.

Method	GR-score 2D		GR-score 3D		Improvement		
	AS 201501 IPv6	AS 200909 IPv4	AS 201501 IPv6	AS 200909 IPv4	AS 201501 IPv6	AS 200909 IPv4	Mean
**RA-LE**	0.02	0.01	0.02	0.01	0.00	0.00	0.00
**RA-ISO**	0.15	0.10	0.10	0.04	-0.05	-0.06	-0.06
**EBC-ncISO**	0.15	0.12	0.10	0.05	-0.05	-0.07	-0.06
**EBC-ISO**	0.17	0.11	0.11	0.04	-0.06	-0.07	-0.07
**RA-ncISO**	0.16	0.14	0.10	0.04	-0.06	-0.10	-0.08
**EBC-LE**	0.02	0.34	0.02	0.01	0.00	-0.33	-0.17
**LE**	0.23	0.28	0.13	0.05	-0.10	-0.23	-0.17
**ncISO**	0.32	0.24	0.16	0.06	-0.16	-0.18	-0.17
**ISO**	0.36	0.30	0.17	0.06	-0.19	-0.24	-0.22

Supplementary Table 11. Comparison of 2D and 3D greedy routing on Internet networks

Two of the Internet networks whose statistics are reported in Supplementary Table 6 have been mapped both in 2D and 3D using the manifold-based coalescent embedding techniques and the greedy routing in the hyperbolic space has been evaluated. The table reports for each method and for each network the GR-score both in 2D and in 3D, in addition to the 3D-improvement. The GR-score is a metric to evaluate the efficiency of the greedy routing, which assumes values between 0, when all the routings are unsuccessful, and 1, when all the packets reach the destination through the shortest path (see Methods for details). Differently from the previous tables, the statistical test has not been performed due to the reduced number of networks considered.

Louvain					Infomap				
**Method**	**Mean NMI 2D**	**Mean NMI 3D**	**Impr.**	**p-value**	**Method**	**Mean NMI 2D**	**Mean NMI 3D**	**Impr.**	**p-value**
**RA-LE**	0.66	0.69	+0.03	0.380	**ISO**	0.67	0.72	+0.05	0.343
**LE**	0.68	0.70	+0.02	0.452	**RA-LE**	0.65	0.67	+0.02	0.429
**ISO**	0.67	0.69	+0.02	0.413	**EBC-ISO**	0.69	0.71	+0.02	0.387
**ncISO**	0.66	0.67	+0.01	0.482	**ncISO**	0.67	0.69	+0.02	0.404
**EBC-LE**	0.64	0.65	+0.01	0.481	**EBC-ncISO**	0.70	0.71	+0.01	0.424
**EBC-ncISO**	0.69	0.70	+0.01	0.450	**LE**	0.68	0.69	+0.01	0.494
**RA-ISO**	0.69	0.69	0.00	0.487	**RA-ncISO**	0.68	0.68	0.00	0.489
**EBC-ISO**	0.71	0.70	-0.01	0.473	**RA-ISO**	0.70	0.69	-0.01	0.441
**RA-ncISO**	0.70	0.68	-0.02	0.453	**EBC-LE**	0.68	0.66	-0.02	0.425

Label Propagation					Walktrap				
**Method**	**Mean NMI 2D**	**Mean NMI 3D**	**Impr.**	**p-value**	**Method**	**Mean NMI 2D**	**Mean NMI 3D**	**Impr.**	**p-value**
**ncISO**	0.59	0.62	+0.03	0.410	**EBC-LE**	0.63	0.66	+0.03	0.436
**EBC-LE**	0.60	0.63	+0.03	0.391	**RA-LE**	0.63	0.64	+0.01	0.492
**RA-ISO**	0.63	0.65	+0.02	0.430	**EBC-ncISO**	0.66	0.67	+0.01	0.469
**LE**	0.62	0.62	0.00	0.491	**ISO**	0.65	0.65	0.00	0.493
**RA-LE**	0.63	0.63	0.00	0.488	**ncISO**	0.64	0.64	0.00	0.505
**RA-ncISO**	0.63	0.62	-0.01	0.471	**RA-ncISO**	0.64	0.64	0.00	0.503
**EBC-ISO**	0.63	0.61	-0.02	0.438	**EBC-ISO**	0.65	0.65	0.00	0.483
**EBC-ncISO**	0.64	0.61	-0.03	0.419	**LE**	0.66	0.65	-0.01	0.474
**ISO**	0.67	0.63	-0.04	0.360	**RA-ISO**	0.65	0.64	-0.01	0.456

Supplementary Table 12. Comparison of 2D and 3D community detection on real networks

The 8 real networks whose statistics are reported in Table 1 have been mapped both in 2D and 3D using the manifold-based coalescent embedding techniques and the community detection has been evaluated exploiting the 2D and 3D hyperbolic distances to weight the input matrix for the four community detection algorithms. The table reports for each method the mean NMI over all the networks both in 2D and in 3D, highlighting the 3D-improvement. NMI is the normalized mutual information and represents the shared information between two distributions, normalized between 0 and 1, where 1 indicates that the communities detected by the algorithm perfectly correspond to the ground truth communities (see Methods for details). The rightmost column of each community detection algorithm shows for each embedding method the p-value of the permutation test for the mean (10000 iterations) performed considering the two vectors of GR-scores related to 2D and 3D.

Supplementary Note 1: Repulsion-Attraction pre-weighting rules

$$x_{ij}^{RA1} = \frac{d_i + d_j + d_i d_j}{1 + CN_{ij}} \quad (1)$$

$$x_{ij}^{RA2} = \frac{1 + e_i + e_j + e_i e_j}{1 + CN_{ij}} \quad (2)$$

$$x_{ij}^{RA3} = \frac{d_i + d_j}{1 + CN_{ij}} \quad (3)$$

$$x_{ij}^{RA4} = \frac{d_i d_j}{1 + CN_{ij}} \quad (4)$$

In the mathematical expressions: x_{ij} is the value of an edge (i, j) in the adjacency matrix; d_i is the degree of the node *i*; e_i is the external degree of the node *i* with respect to node *j* (links to nodes that are neither common neighbours nor the node *j*); CN_{ij} are the common neighbours of nodes *i* and *j*.

Supplementary Discussion

Greedy routing performance in synthetic and real networks

In this section, we would like to explain the reason why RA-ncMCE resulted the best performing among the coalescent embedding methods on the greedy routing tests.

Firstly, we would like to underline the relevant increase of performance obtained by ncMCE when the RA pre-weighting is applied, which confirms the efficacy of the RA rule. The RA pre-weighting is very effective to suggest the hidden geometry to extract the MST on the basis of which the distances that are collected in the MC-kernel are approximated (see Methods for details). In fact, ncMCE alone offers a very poor performance in GR, while RA-ncMCE provides top performance between the coalescent embedding techniques.

Secondly, the fact that ncMCE is performing better than MCE in problems of network embedding was also proved and discussed in a previous publication[2], and it is related with the effect of the kernel centring procedure, therefore we will not discuss further here.

At last, a theoretical digression is necessary in order to explain why RA-ncMCE performed better than the manifold-based coalescent embedding techniques. The embedding methods based on matrix factorization are all global methods because they exploit an *NxN* matrix decomposition[3]. However, they mainly work in different ways. LE is a neighbourhood-

preserving global method, in fact, the Laplacian matrix to which eigen-decomposition is applied only contains information about connected nodes, therefore it infers angular coordinates that give preference to put connected nodes closer. ISO and ncISO, on the contrary, belong to the class of global methods that aim to preserve the global topology of the neighbourhood graph that approximates the hidden manifold geometry, therefore they do not concentrate exclusively on the accurate preservation of connected points at close angular coordinates. Indeed, they apply singular value decomposition to a distance kernel, which contains information about both connected and disconnected nodes. It means that they attempt to preserve geometry at all scales, therefore introducing and distributing the error at all the scales. ISO procedure does not give preference to an accurate preservation of connected points that is a necessary procedure for effective GR but, on the other hand, tries to fulfil the second important condition to put disconnected points far in the angular coordinates. ncMCE is a global method that preserves a locally-reconstructed (by means of MST) global geometry by overestimating distances between disconnected nodes. Since the MC-kernel is obtained by computing all pairwise transversal distances over the MST, in practice distances for both connected and disconnected nodes are approximated. However, the distances of connected nodes will be 'fairly' estimated, while the ones of disconnected nodes will be overrated. In conclusion, minimum curvilinearity, which is the mechanism of generation of the MC-kernel, favours inference of kernel distances that preserve connected nodes close in the angular coordinates, and push disconnected nodes far apart in the angular coordinates.

Community detection on real networks

This section is intended to provide further discussions about the results on the community detection application. As first, we would like to comment the fact that, differently from the embedding evaluation on the synthetic networks, in community detection on real networks ncISO-based and MCE-based coalescent embedding techniques are significantly better than LE-based methods (Table 1-2 and Supplementary Table 1-4). Not to be overlooked, EBC-ncISO-EA is the only method that improves with respect to all the four unweighted algorithms. As expected, this finding suggests that the results obtained on synthetic networks are indicative but should be taken with caution. Real networks might have a geometry that is even more tree-like and hyperbolic than the one hypothesized by the PSO model (for this reason MCE-based techniques can perform better on real networks), and although the topology of real networks is certainly conditioned by the hyperbolic geometry this is however one of the factors that shape their structure. On the other side, good results are achieved also for networks with out of range

γ values such as Opsahl_11. Since it has been demonstrated that a scale-free degree distribution is a necessary condition for hyperbolic geometry[4], this result demonstrates that the methods can reach good performances also for networks whose latent geometry might be weakly hyperbolic.

As second point, we want to highlight that also in community detection simulations on real world networks the contribution offered by EA is evident. Except for Label propagation where the EA and non-EA methods show a mixed ranking (Supplementary Table 3), in Louvain, Infomap and Walktrap the EA-based coalescent embedding techniques offer the best performance (Supplementary Table 1, 2 and 4), confirming that the adjustment of local embedding uncertainty can be crucial for effective coalescent embedding also in real applications. We note that Louvain was the only method for which the improvement in community detection was higher using not only the geometrical information between the connected nodes but also between disconnected nodes. Supplementary Table 1 and 5 show the different results using either a kernel or a weighted network in input. For the other community detection algorithms the results are not reported since the usage of a kernel led to totally wrong predictions.

In order to test the coalescent embedding methods on real networks of larger size, the community detection has been performed also on Internet networks ranging from 5000 up to 37000 nodes, where each node represents an Autonomous System and the connections indicate the IPv4 or IPv6 topology (see dataset description for details). For Louvain, the processing of the kernel matrix for large networks resulted to be too computationally expensive from the point of view of the memory requirements, therefore the results are not reported. For Infomap and Walktrap, most of the embedding methods obtained the same performance as the unweighted variant, and also the other techniques did not show a big deviation from that reference (Supplementary Table 6 and 8). However, for Infomap, many MCE-based approaches offered a small increase of performance and the only improving method for Walktrap is also MCE-based. Differently, for Label propagation the unweighted variant obtained a very low result, therefore there was a higher margin of improvement and the usage of the geometrical information led to a significant boosting (Supplementary Table 7).

Beyond the two-dimensional space

Before starting with the analysis of the results, there is the need to discuss a preliminary point. Coalescent embedding at the moment includes two different types, the manifold-based (LE, ISO, ncISO) and the Minimum Curvilinearity (MCE, ncMCE) approaches. The latter ones are

fundamentally different, since they learn the nonlinear similarities by means of MST and linearize the hidden pattern providing a hierarchical-based mapping. Therefore, on one side we are seeking to exploit an additional dimension of embedding, on the other side the power of the Minimum Curvilinearity methods is the compression of information in a single dimension: it is evident by definition that it would be a contradiction to adopt them for this investigation. Furthermore, while the rearrangement of the linearized similarities over a circumference remains intuitive, it is not trivial to find a meaningful way for reorganizing the linearized pattern over a sphere. Note that, for analogous reasons, also the equidistant adjustment is not adopted. On the contrary, the manifold-based approaches offer less compression capabilities and therefore do not exclude the presence of potentially useful information in the third dimension. Moreover, since the hidden similarity pattern can remain nonlinear also in the embedded space, the similarities can be directly accommodated to the sphere without the need of any particular reorganization, as it happens using two dimensions.

Supplementary Table 9 reports the mean difference between the GR scores of the 3D versus 2D greedy routing performed on the PSO networks embedded in the hyperbolic space, where the mean is taken over all the parameter combinations. The table highlights a small even though significant (p-value < 0.001) improvement obtained with the addition of the third dimension in ISO and ncISO. However, there is no significant improvement for the other methods, and for the LE-based approaches there is even a small decline. Supplementary Table 10 reports the mean difference between the GR scores of the 3D versus 2D greedy routing performed on the real small-size networks embedded in the hyperbolic space, where the mean is taken over all the networks. The table shows that, except for EBC-LE, the methods slightly improve the performance with a gain in NMI up to 0.05, but not significantly. Supplementary Table 11 reports the difference between the GR scores of the 3D versus 2D greedy routing performed on two AS networks embedded in the hyperbolic space, one middle size and one large size. The table underlines the presence of a general performance decrease with the addition of the third dimension.

Supplementary Table 12 reports the mean difference between the NMI scores of the 3D versus 2D community detection performed on the real small-size networks embedded in the hyperbolic space, where the mean is taken over all the networks. The table highlights that for each community detection method the coalescent embedding approaches oscillate between a small increase and a little decrease of performance using the third dimension, but the difference is not significant in any case.

Notes on pre-weighting, rich-clubness and angular adjustment

The proposed class of coalescent embedding algorithms includes several variants and, except for the different machine learning techniques, the variations are given by the pre-weighting and the angular adjustment, whose contribution will be now discussed.

Out of question is the positive effect of the pre-weighting on the embedding accuracy. All the simulations highlighted that suggesting topological similarities between the connected nodes makes the inference of the coordinates more precise and leads to remarkable improvements in performance (Supplementary Fig. 2-6). Furthermore, both the local-based RA rule and the global-based EBC rule resulted to be effective. Since there was not a unique possible mathematical formulation of the RA formula, we tested four variants differing in the way in which the degrees of the connected nodes are combined, the mathematical expressions are shown in Supplementary Note 1 and the results in Supplementary Fig. 23. The variant RA_1 gave the best results for the embedding of small-size PSO networks, whereas the variant RA_2 for the large-size PSO networks, although the performance is very similar for all of them. Therefore, we here propose to adopt both the versions RA_1 and RA_2, as reported in Fig. 2. Since in most of the simulations the networks are small and the results of the two variants are very close, for sake of brevity we showed all the other results only for RA_1. We let notice that from a theoretical point of view the formula RA_2 is more correct, in fact it conceptually splits the neighbours of the adjacent nodes in two non-overlapping subsets: the neighbours not in common (external degree), responsible for the repulsive part, and the common neighbours, determining the attractive part. In the other formulas, instead, the numerator considers not the external degree but the degree of the adjacent nodes, which includes also the common neighbours. However, on the tested networks it emerges that this conceptual difference does not lead to a substantial performance improvement, but it might play an important role for networks of larger size.

It might be argued that the repulsion between high (external) degree nodes implied by the RA rule is in contrast with the existence of rich-clubs. In rich-club networks, high degree nodes (hubs) tend to connect each other[5]. However, we would like to clarify that the repulsive part of the rule is not suggesting that nodes with high (external) degree tend to be disconnected. It suggests that they tend to dominate geometrically distant regions, which does not exclude their connectivity and therefore it should not be theoretically in contrast to the existence of rich-clubs. In order to prove this point by experiments, we started performing a statistical test for rich-clubness[1] on the PSO networks used for the previous simulations, the p-values are reported in Supplementary Fig. 25. The statistical test highlights that for most of the parameter

combinations, in particular for m = [4, 6] and N = [500, 1000] the networks present a significant rich-club, whereas for more sparse (m = 2) and small networks (N = 100) in general there is not a significant rich-club. This is in agreement with the network growing procedure explained by the PSO model. In fact, the high degree nodes are the first ones to be born in the network and they are expected to connect to around m of the older nodes[6]. Therefore for higher m the rich nodes have higher probability to create a club. Looking at Supplementary Fig. 2-6, it is evident that for networks with m = [4, 6], which are significantly rich-club, the methods using the RA pre-weighting rule do not have any particular decrease in performance, they still provide a very high improvement with respect to the unweighted variant, as for networks with m = 2 that do not contain a significant rich-club. We therefore conclude that the RA pre-weighting rule can be adopted regardless of the rich-clubness of a network.

If on one side there are no doubts about the essentialness of the pre-weighting, a discussion is required on the contribution of the equidistant adjustment. In fact, the significant improvement obtained using EA on PSO networks up to 1000 nodes (Fig. 3 and Supplementary Fig. 7) might be due to overfitting to the PSO model, since the angular coordinates are randomly generated by a uniform sampling. Interestingly, Fig. 5 suggests that for PSO networks of size 30000 the EA contribution vanishes. The reason is that with bigger networks the high number of nodes tends to densely and more uniformly cover the angular range hence the non-EA embedding already arranges the nodes in an almost exactly equidistant way. Looking at the community detection application, we noticed that for three out four algorithms (Louvain, Infomap and Walktrap) the EA-based methods obtained in general higher performances than the respective non-EA versions, suggesting that the adjustment of local embedding uncertainty can be effective in real applications. As last, we checked the contribution of the equidistant adjustment on greedy routing. As reported in Supplementary Fig. 20, the equidistant adjustment offered an overall improvement for the greedy routing on PSO networks and a decrease in performance on real networks, although small. To conclude, due to the variable contribution given by the equidistant adjustment, we propose it as a valid alternative to take into consideration, even if it does not represent always the best option. New methods of angular adjustment should be investigated in future studies.

Supplementary References

1. Muscoloni, A. & Cannistraci, C. V. Rich-clubness test: how to determine whether a complex network has or doesn't have a rich-club? *arXiv:1704.03526v1 [physics.soc-ph]* (2017).
2. Cannistraci, C. V., Alanis-Lobato, G. & Ravasi, T. Minimum curvilinearity to enhance topological prediction of protein interactions by network embedding. *Bioinformatics* **29,** 199–209 (2013).
3. Saul, L. K. L. & Roweis, S. S. T. Think globally, fit locally: unsupervised learning of low dimensional manifolds. *J. Mach. Learn. Res.* **4,** 119–155 (2003).
4. Krioukov, D., Papadopoulos, F., Kitsak, M., Vahdat, A. & Boguñá, M. Hyperbolic geometry of complex networks. *Phys. Rev. E - Stat. Nonlinear, Soft Matter Phys.* **82,** 36106 (2010).
5. Zhou, S. & Mondragón, R. J. The Rich-Club Phenomenon in the Internet Topology. *IEEE Commun. Lett.* **8,** 180–182 (2004).
6. Papadopoulos, F., Kitsak, M., Serrano, M. A., Boguna, M. & Krioukov, D. Popularity versus similarity in growing networks. *Nature* **489,** 537–540 (2012).
